# Need for Cognition is associated with a preference for higher task load in effort discounting

**DOI:** 10.1038/s41598-023-44349-3

**Published:** 2023-11-09

**Authors:** Josephine Zerna, Christoph Scheffel, Corinna Kührt, Alexander Strobel

**Affiliations:** Chair for Differential and Personality Psychology, Technische Universität Dresden, Zellescher Weg 17, Dresden, 01069 Germany

**Keywords:** Human behaviour, Reward, Decision

## Abstract

When individuals set goals, they consider the subjective value (SV) of the anticipated reward and the required effort, a trade-off that is of great interest to psychological research. One approach to quantify the SVs of levels of difficulty of a cognitive task is the Cognitive Effort Discounting Paradigm by Westbrook and colleagues (2013). However, it fails to acknowledge the highly individual nature of effort, as it assumes a unidirectional, inverse relationship between task load and SVs. Therefore, it cannot map differences in effort perception that arise from traits like Need for Cognition, since individuals who enjoy effortful cognitive activities likely do not prefer the easiest level. We replicated the analysis of Westbrook and colleagues with an adapted version, the Cognitive and Affective Discounting (CAD) Paradigm. It quantifies SVs without assuming that the easiest level is preferred, thereby enabling the assessment of SVs for tasks without objective order of task load. Results show that many of the 116 participants preferred a more or the most difficult level. Variance in SVs was best explained by a declining logistic contrast of the $n$-back levels and by the accuracy of responses, while reaction time as a predictor was highly volatile depending on the preprocessing pipeline. Participants with higher Need for Cognition scores perceived higher $n$-back levels as less effortful and found them less aversive. Effects of Need for Cognition on SVs in lower levels did not reach significance, as group differences only emerged in higher levels. The CAD Paradigm appears to be well suited for assessing and analysing task preferences independent of the supposed objective task difficulty.

**Protocol registration**The stage 1 protocol for this Registered Report was accepted in principle on August 19, 2022. The protocol, as accepted by the journal, can be found at: 10.17605/OSF.IO/CPXTH.

## Introduction

In everyday life, effort and reward are closely intertwined^1^. With each decision a person makes, they have to evaluate whether the effort required to reach a goal is worth being exerted, given the reward they receive when reaching the goal. A reward is subjectively more valuable if it is obtained with less effort, so the required effort is used as a reference point for estimating the reward value^1^. However, the cost of the effort itself is also subjective, and research has not yet established which function best describes the relationship between effort and cost^2^. Investigating effort and cost is challenging because “effort is not a property of the target task alone, but also a function of the individual’s cognitive capacities, as well as the degree of effort voluntarily mobilized for the task, which in turn is a function of the individual’s reward sensitivity” (p. 209)^2^.

One task that is often used to investigate effort is the $n$-back task, a working memory task in which a continuous stream of stimuli, e.g. letters, is presented on screen. Participants indicate via button press whether the current stimulus is the same as $n$ stimuli before, with $n$ being the level of difficulty between one and six^3^. The $n$-back task is well suited to investigate effort because it is an almost continuous manipulation of task load as has been shown by monotonic increases in error rates, reaction times^4^, and brain activity in areas associated with working memory^5,6^. However, its reliability measures are mixed, and associations of $n$-back performance and measures such as executive functioning and fluid intelligence are often inconsistent^4^.

A way to quantify the subjective cost of each $$n$$-back level has been developed by Westbrook, Kester, and Braver^7^, called the Cognitive Effort Discounting Paradigm (COG-ED). First, the participants complete the $$n$$-back levels to familiarize themselves with the task. Then, 1-back is compared with each more difficult level by asking the participants to decide between receiving a fixed 2$ for the more difficult level or the flexible starting value of 1$ for 1-back. If they choose the more difficult level, the reward for 1-back increases by 0.50$, if they choose 1-back, it decreases by 0.50$. This is repeated five more times, with each adjustment of the 1-back reward being half of the previous step, while the reward for the more difficult level remains fixed at 2$. The idea is to estimate the point of subjective equivalence, i.e., the monetary ratio at which both offers are equally preferred^7^. The subjective value (SV) of each more difficult level is then calculated by dividing the final reward value of 1-back by the fixed 2$ reward. Westbrook et al.^7^ used these SVs to investigate inter-individual differences in effort discounting. Younger participants showed lower effort discounting, i.e., they needed a lower monetary incentive for choosing the more difficult levels over 1-back.

The individual degree of effort discounting in the study by Westbrook et al.^7^ was also associated with the participants’ scores in Need for Cognition (NFC), a personality trait describing an individual’s tendency to actively seek out and enjoy effortful cognitive activities^8^. Westbrook et al.^7^ conceptualized NFC as a trait measure of effortful task engagement, providing a subjective self-report of effort discounting for each participant which could then be related to the SVs as an objective measure of effort discounting. On the surface, this association stands to reason, as individuals with higher NFC are more motivated to mobilize cognitive effort because they perceive it as intrinsically rewarding. Additionally, it has been shown that individuals avoid cognitive effort only to a certain degree, possibly to retain a sense of self-control^9^, a trait more prominent in individuals with high NFC^10–12^. However, the relation of NFC and SVs might be confounded, since other studies utilizing the COG-ED paradigm found the association of NFC and SVs to disappear after correcting for performance^13^ or found no association of NFC and SVs at all^14^. On the other hand, task load has been shown to be a better predictor of SVs than task performance^7,15,16^, so more research is needed to shed light on this issue.

With the present study, we alter one fundamental assumption of the original COG-ED paradigm: That the easiest $n$-back level has the highest SV. We therefore adapted the COG-ED paradigm in a way that allows the computation of SVs for different $n$-back levels without presuming that all individuals inherently prefer the easiest level. Since we also aim to establish this paradigm for the assessment of tasks with no objective task load, e.g., emotion regulation tasks^17^, we call it the Cognitive and Affective Discounting Paradigm (CAD). In the present study, we validated the CAD paradigm by conceptually replicating the findings of Westbrook et al.^7^. Additionally, we compared the effort discounting behavior of participants regarding the $n$-back task and an emotion regulation task. The full results of the latter are published in a second Registered Report^17^. The COG-ED paradigm has been applied to tasks in different domains before, showing that SVs across task domains correlate^14^, but these tasks had an objective order of task load, which is not the case for the choice of emotion regulation strategies or other paradigms where there is no objective order of task load.

Our hypotheses were derived from the results of Westbrook et al.^7^. As a manipulation check, we hypothesized that with increasing $n$-back level the (1a) the signal detection parameter $d'$ declines, while (1b) reaction time and (1c) perceived task load increase. Regarding the associations of task load and effort discounting we hypothesized that (2a) SVs decline with increasing $n$-back level, and (2b) they do so even after controlling for declining task performance. And finally, we hypothesized that the CAD paradigm can show inter-individual differences in effort discounting, such that participants with higher NFC have (3a) lower SVs for 1-back but higher SVs for 2- and 3-back, (3b) lower perceived task load across all levels, and (3c) higher aversion against 1-back but lower aversion against 2- and 3-back. Each hypothesis is detailed in the Design Table in the Supplementary Material.

## Methods

We report how we determined our sample size, all data exclusions (if any), all manipulations, and all measures in the study^cf.^^18^. The paradigm was written and presented using *PsychoPy*^19^. We used *R* (Version 4.2.0)^20^ with *R Studio* (Version 2022.12.0)^21^ with the main packages *papaja* (Version 0.1.1)^22^, *afex* (Version 1.2-1)^23^, and *BayesFactor* (Version 0.9.12-4.4)^24^ for all our analyses.

### Ethics information

The study protocol complies with all relevant ethical regulations and was approved by the ethics committee of the Technische Universität Dresden (reference number SR-EK-50012022). Prior to testing, written informed consent was obtained. Participants received 24€ in total or course credit for participation.

### Design

#### CAD Paradigm

**Figure 1:**
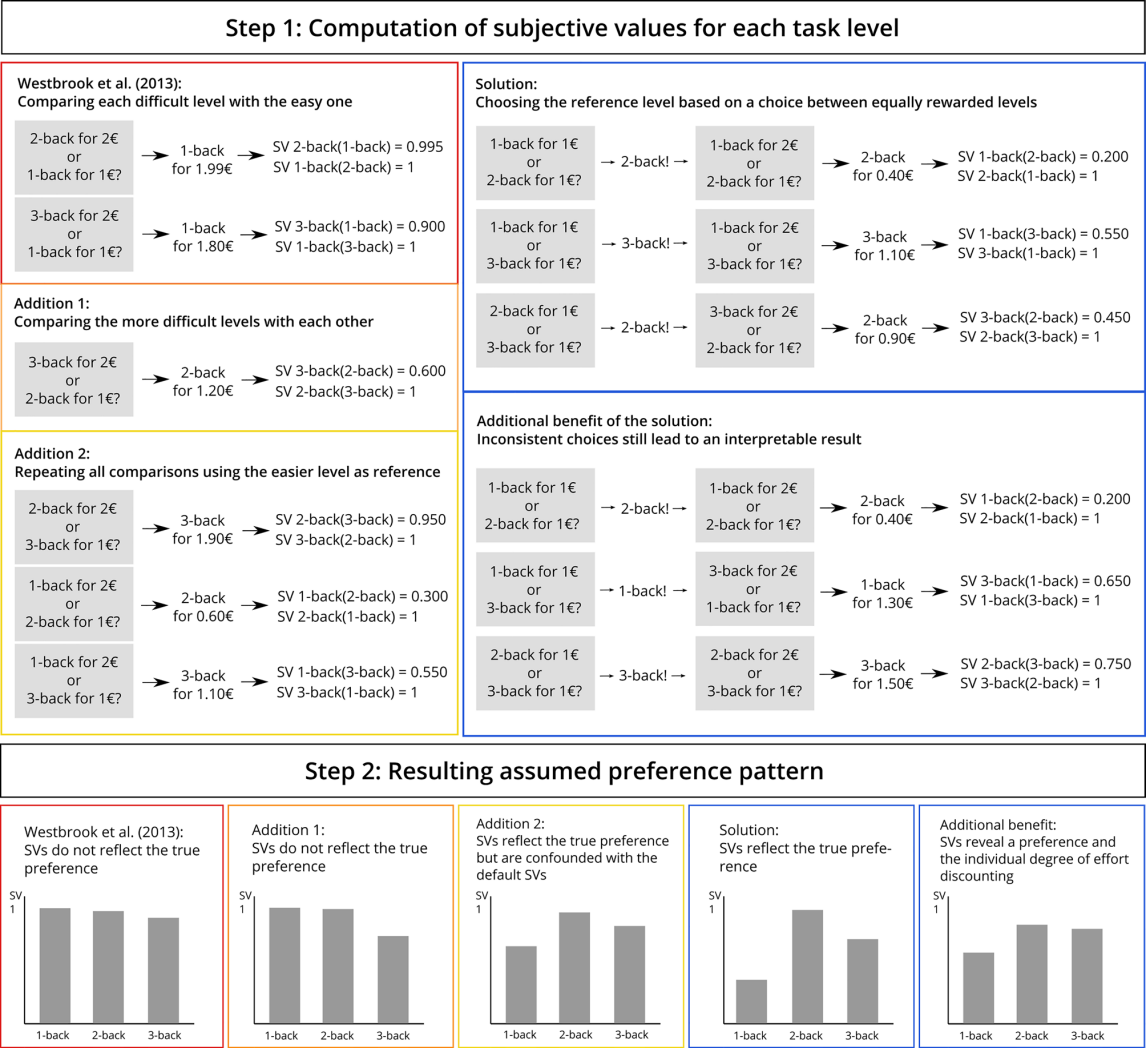
An example for subjective values for an n-back task with three levels, returned by different modifications of the COG-ED paradigm for a hypothetical participant with the true preference 2-back > 3-back > 1-back. The grey boxes are the choice options shown to the participant. The participant's final reward value of the flexible level is displayed after the first arrow. The resulting subjective value of each level is displayed after the second arrow, in the notation "SV 3-back(1-back)" for the subjective value of 3-back when 1-back is the other choice. The Solution and Additional Benefit panel follow the same logic, but are preceded by a choice between equal rewards, and the participant's first choice indicated by an exclamation mark. Figure available at osf.io/vnj8x/, under a CC-BY-4.0 license.

Figure 1 illustrates how different modifications of the COG-ED paradigm^7^ return SVs that do or do not reflect the true preference of a hypothetical participant, who likes 2-back most, 3-back less, and 1-back least (for reasons of clarity there are only three levels in the example). The COG-ED paradigm, which compares every more difficult level with 1-back sets the SV of 1-back to 1, regardless of the response pattern. Adding a comparison of the more difficult levels with each other allows the SVs of those two levels to be more differentiated, but leaves the SV of 1-back unchanged. Adding those same pairs again, but with the opposite assignment of fixed and flexible level, does approach the true preference, but has two disadvantages. First, the SVs are still quite alike across levels due to the fact that every more difficult level has only been compared with the easiest level, and second, having more task levels than just three would lead to an exponential increase in comparisons. Therefore, the solution lies in reducing the number of necessary comparisons by presenting only one effort discounting round for each possible pair of levels after determining for each pair which level should be fixed and which should be flexible. This is determined by presenting each possible pair of levels on screen with the question “Would you prefer 1€ for level A or 1€ for level B?”. Participants respond by clicking the respective on-screen button. Each pair is presented three times, resulting in 18 presented pairs, which are fully randomized in order and in the assignment of which level is on the left or right of the screen. For each pair, the level that was chosen by the participant at least two out of three times will be used as the level with a flexible value, which starts at 1€ and changes in every iteration. The other level in the pair will be set to a fixed value of 2€. Then, the effort discounting sensu Westbrook et al.^7^ begins, but with all possible pairs and with the individually determined assignment of fixed and flexible level. The order in which the pairs are presented is fully randomized, and each pair goes through all iteration steps of adding/subtracting 0.50€, 0.25€, 0.13€, 0.06€, 0.03€, 0.02€ to/from the flexible level’s reward (each adjustment half of the previous one, rounded to two decimals) before moving on to the next one. This procedure allows to compute SVs based on actual individual preference instead of objective task load. For each pair, the SV of the flexible level is 1, as it was preferred when faced with equal rewards, and the SV of the fixed level is the final reward of the flexible level divided by 2€. Each level’s “global” SV is calculated as the mean of this level’s SVs from all pairs in which it appeared. If the participant has a clear preference for one level, this level’s SV will be 1. If not, then no level’s SV will be 1, but each level’s SV can still be interpreted as an absolute and relative value, so each participant’s effort discounting behaviour can still be quantified. The interpretation of SVs in Westbrook et al.^7^ was “The minimum relative reward required for me to choose 1-back over this level”. So if the SV of 3-back was 0.6, the participant would need to be rewarded with at least 60 % of what they are being offered for doing 3-back to do 1-back instead, forgoing the higher reward for 3-back. In this study, the SV can be interpreted as “The minimum relative reward required for me to choose any other level over this level”. Therefore, an SV of 1 indicates that this level is preferred over all others, while SVs lower than 1 indicate that in at least one pair, a different level was preferred over this one.

#### Study procedure

Healthy participants aged 18 to 30 years were recruited using the software *ORSEE*^25^. Participants completed the personality questionnaires online and then visited the lab for two sessions one week apart. NFC was assessed using the 16-item short form of the Need for Cognition Scale^26,27^. Responses to each item (e.g., “Thinking is not my idea of fun”, recoded) were recorded on a 7-point Likert scale. The NFC scale shows comparably high internal consistency (Cronbach’s $\alpha >0.80$)^27,28^. Several other personality questionnaires were used in this study but are the topic of the Registered Report for the second lab session^17^. A full list of measures can be found in our Github repository. In the first session, participants provided informed consent and demographic data before completing the computer-based paradigm. The paradigm started with the $n$-back levels one to four, presented sequentially with two runs per level, consisting of 64 consonants (16 targets, 48 non-targets) per run. The levels were referred to by color (1-back: black, 2-back: red, 3-back: blue, 4-back: green) to avoid anchor effects in the effort discounting procedure. To assess perceived task load, we used the 6-item NASA Task Load Index (NASA-TLX)^29^, where participants evaluate their subjective perception of mental load, physical load, effort, frustration, performance, and time pressure during the task on a 20-point scale. At the end of each level, participants filled out the NASA-TLX on a tablet, plus an item with the same response scale, asking them how aversive they found this $n$-back level. After the $n$-back task, participants completed the CAD paradigm on screen and were instructed to do so as realistically as possible, even though the displayed rewards were not paid out on top of their compensation. They were told that one of their choices would be randomly picked for the final run of $n$-back. However, this data was not analyzed as it only served to incentivise truthful behavior and to stay close to the design of Westbrook et al.^7^. After the CAD paradigm, participants filled out a short questionnaire on the tablet, indicating whether they adhered to the instructions (yes/no) and what the primary motivation for their decisions during the effort discounting procedure was (avoid boredom/relax/avoid effort/seek challenge/other).

The second session consisted of an emotion regulation task with negative pictures and the instruction to suppress facial reactions, detach cognitively from the picture content, and distract oneself, respectively. The paradigm followed the same structure of task and effort discounting procedure, but participants could decide which strategy they wanted to reapply in the last block. Study data was collected and managed using REDCap electronic data capture tools hosted at Technische Universität Dresden^30,31^.

### Sampling plan

Sample size determination was mainly based on the results of the analyses of Westbrook et al.^7^ (see Design Table in the Supplementary Material). The hypothesis that yielded the largest necessary sample size was a repeated measures ANOVA with within-between interaction of NFC and $n$-back level influencing SVs. Sample size analysis with G*Power^32,33^ indicated that we should collect data from at least 72 participants, assuming $\alpha =0.05$ and $\beta =0.95$. However, the sample size analysis for the hypotheses of the second lab session revealed a larger necessary sample size of 85 participants to find an effect of $d=-0.32$ of emotion regulation on facial muscle activity with $\alpha =0.05$ and $\beta =0.95$. To account for technical errors, noisy physiological data, or participants who indicate that they did not follow the instructions, we aimed to collect about $50\%$ more data sets than necessary, $N=120$ in total.

### Analysis plan

Data collection and analysis were not performed blind to the conditions of the experiments. We excluded the data of a participant from all analyses, if the participant stated that they did not follow the instructions, if the investigator noted that the participant misunderstood the instructions, or if the participant withdrew their consent. No data was replaced. The performance measure $d'$ was computed as the difference of the *z*-transformed hit rate and the *z*-transformed false alarm rate^34^. Reaction time (RT) data was trimmed by excluding all trials with responses faster than 100 ms, as the relevant cognitive processes cannot have been completed before^35,36^. Aggregated RT values were described using the median and the median of absolute deviation ($MAD$) as robust estimates of center and variability, respectively^37^. Error- and post-error trials were excluded, because RT in the latter is longer due to more cautious behavior^38,39^. To test our hypotheses, we performed a series of rmANOVAs and an MLM with orthogonal sum-to-zero contrasts in order to meaningfully interpret results^40^.

#### Manipulation check

Declining performance was investigated by calculating an rmANOVA with six paired contrasts comparing $d'$ between two levels of 1- to 4-back at a time. Another rmANOVA with six paired contrasts was computed to compare the median RT between two levels of 1- to 4-back at a time. To investigate changes in NASA-TLX ratings, six rmANOVAs were computed, one for each NASA-TLX subscale, and each with six paired contrasts comparing the ratings between two levels of 1- to 4-back at a time.

#### Subjective values

For each effort discounting round, the SV of the fixed level was calculated by adding or subtracting the last adjustment of 0.02€ from the last monetary value of the flexible level, depending on the participant’s last choice, and dividing this value by 2€. This yielded an SV between 0 and 1 for the fixed compared with the flexible level, while the SV of the flexible level was 1. The closer the SV of the fixed level is to 0, the stronger the preference for the flexible level. All SVs of each level were averaged to compute one “global” SV for each level. An rmANOVA with four different contrasts were computed to investigate the association of SVs and the $n$-back levels: Declining linear (3, 1, − 1, − 3), ascending quadratic (− 1, 1, 1, − 1), declining logistic (3, 2, − 2, − 3), and positively skewed normal (1, 2, − 1, − 2) (Supplementary Fig. S1). Depending on whether the linear or one of the other three contrasts fit the curve best, we applied a linear or nonlinear multi-level model in the next step, respectively.

To determine the influence of task performance on the association of SVs and $n$-back level, we performed MLM. We applied restricted maximum likelihood (REML) to fit the model. As an effect size measure for random effects we first calculated the intraclass correlation (ICC), which displays the proportion of variance that is explained by differences between persons. Second, we estimated a random slopes model of $n$-back level (level 1, fixed, and random factor: 0-back, 1-back, 2-back, 3-back) predicting SV nested within subjects. As Mussel et al.^41^ could show, participants with high versus low NFC not only have a more shallow decline in performance with higher $n$-back levels, but show a demand-specific increase in EEG theta oscillations, which has been associated with mental effort. We controlled for performance, i.e., $d'$ (level 1, fixed factor, continuous), median RT (level 1, fixed factor, continuous) in order to eliminate a possible influence of declining performance on SV ratings.

$$\begin{aligned} SV \sim level\ + d' + median RT + \left(level|subject\right) \end{aligned}$$

Level-1-predictors were centered within cluster as recommended by Enders & Tofighi^42^. By this, the model yields interpretable parameter estimates. If necessary, we adjusted the optimization algorithm to improve model fit. We visually inspected the residuals of the model for evidence to perform model criticism. This was done by excluding all data points with absolute standardized residuals above 3 SD. As effect size measures, we calculated pseudo $R^{2}$ for our model and $f^{2}$ to estimate the effect of $n$-back level according to Lorah^43^.

The association of SVs and NFC was examined with an rmANOVA. We subtracted the SV of 1- from 2-back and 2- from 3-back, yielding two SV difference scores per participant. The sample was divided into participants with low and high NFC using a median split. We then computed an rmANOVA with the within-factor $n$-back level and the between-factor NFC group to determine whether there is a main effect of level and/or group, and/or an interaction between level and group on the SV difference scores. Post-hoc tests were computed depending on which effect reached significance at $p<0.01$. To ensure the validity of this association, we conducted a specification curve analysis^44^, which included 63 possible preprocessing pipelines of the RT data. These pipelines specify which transformation was applied (none, log, inverse, or square-root), which outliers were excluded (none, 2, 2.5, or 3 $MAD$ from the median, RTs below 100 or 200 ms), and across which dimensions the transformations and exclusions were applied (across/within subjects and across/within $n$-back levels). The rmANOVA was run with each of the 63 pipelines, which also included our main pipeline (untransformed data, exclusion of RTs below 100 ms). The ratio of pipelines that lead to significant versus non-significant effects provides an indication of how robust the effect actually is.

The association of subjective task load with NFC was examined similarly. We calculated NASA-TLX sum scores per participant per level, computed an rmANOVA with the within-factor $n$-back level and the between-factor NFC group, and applied post-hoc tests based on which effect reached significance at $p<0.01$. And the association of subjective aversiveness of the task with NFC was examined with difference scores as well, since we expected this curve to mirror the SV curve, i.e. as the SV rises, the aversiveness declines, and vice versa. We subtracted the aversiveness ratings of 1- from 2-back and 2- from 3-back, yielding two aversiveness difference scores per participant. Then, we computed an rmANOVA with the within-factor $n$-back level and the between-factor NFC group, and applied post-hoc tests based on which effect reached significance at $p<0.01$.

The results of each analysis was assessed on the basis of both $p$-value and the Bayes factor $BF_{10}$, calculated with the *BayesFactor* package^24^ using the default prior widths of the functions *anovaBF*, *lmBF* and *ttestBF*. We considered a $BF_{10}$ close to or above 3/10 as moderate/strong evidence for the alternative hypothesis, and a $BF_{10}$ close to or below 0.33/0.10 as moderate/strong evidence for the null hypothesis^45^.

### Pilot data

The sample of the pilot study consisted of $N=15$ participants (53.3% female, $M=24.43$ ($SD=3.59$) years old). One participant’s data was removed because they misunderstood the instruction. Due to a technical error the subjective task load data of one participant was incomplete, so the hypotheses involving the NASA-TLX were analyzed with $n=14$ data sets. The results showed increases in subjective and objective task load measures with higher $n$-back level. Importantly, SVs were lower for higher $n$-back levels, but not different between 1- and 2-back, which shows that the easiest level is not universally preferred. The MLM revealed $n$-back level as a reliable predictor of SV, even after controlling for declining task performance ($d'$ and median RT). NASA-TLX scores were higher with higher $n$, and lower for the group with lower NFC scores, but NFC and $n$-back level did not interact. All results are detailed in the Supplementary Material.

## Results

### Adjustments for stage 2

There were two necessary adjustments of the methods. First, we failed to update the necessary sample size after the analyses changed with the first review round. Instead of the 72 subjects stated above, the largest minimum sample size was actually 53 subjects (see hypothesis 1b in the Design Table in the Supplementary Material). And secondly, we changed to which hypothesis we applied the specification curve analysis (SCA). In the initial Stage 1 submission, we had applied it to the MLM of hypothesis 2b, which at this point included NFC as a predictor. Following the advice of the reviewers, we removed NFC from the MLM, and analyzed NFC in an rmANOVA (hypothesis 3a) instead. Since NFC was of great interest to us, we decided to apply the SCA to hypothesis 3a rather than 2b to provide a measure of robustness. However, hypothesis 3a does not contain any RT data, so the SCA is only useful for the MLM in hypothesis 2b. Therefore, we applied it to the MLM. The final adjustment was made during the Stage 2 revision. A fellow researcher made us aware that by using the z-transformed hit and false alarm rates for the computation of $d'$, the mean of $d'$ would be approximately 0 for each $n$-back level by design. Consequently, $d'$ could not show changes across $n$-back levels in the manipulation check and would likely yield different results in the MLM. Therefore, we computed $d'$ with unstandardized hit and false alarm rates. We would like to thank Georgia Clay for pointing us to this fallacy.

### Sample

Data was collected between the 16th of August 2022 and the 3rd of February 2023. Of the $N=176$ participants who filled out the NFC questionnaire, $n=124$ completed the first lab session. Based on the experimenters’ notes, we excluded the data of seven participants from analysis for misunderstanding the instruction of the $n$-back task, and the data of one participant who reported that they confused the colours of the levels during effort discounting. Our final data set therefore included $N=116$ participants (83.60% female, $M \pm SD=22.4 \pm 3$) years old), which is 2.2 times more than what the highest sample size calculation required.

### Manipulation checks

We used rmANOVAs to investigate whether objective performance measures and subjective task load measures changed across $n$-back levels. For each rmANOVA we report the generalized eta squared $\hat{\eta }^2_G$, which estimates the effect size in analyses that contain both manipulated and non-manipulated terms. In line with hypothesis H1a, the performance measure $d'$ decreased across $n$-back levels ($F(2.74, 315.51) = 197.18$, $p < .001$, $\hat{\eta }^2_G = .464$, 90% CI $[.403, .516]$, $\text{BF}_{\text {10}} = 1.56 \times 10^{101}$). It decreased more strongly from 2- to 3-back ($t(345) = 10.41$, $p_{Tukey(4)} < .001$, $\text{BF}_{\text {10}} = 3.71 \times 10^{23}$) than from 1- to 2-back ($t(345) = 4.31$, $p_{Tukey(4)} < .001$, $\text{BF}_{\text {10}} = 93, 954.04$) or 3- to 4-back ($t(345) = 7.18$, $p_{Tukey(4)} < .001$, $\text{BF}_{\text {10}} = 2.45 \times 10^{14}$). Similarly, the median RT increased across $n$-back levels ($F(2.46, 283.05) = 98.67$, $p < .001$, $\hat{\eta }^2_G = .192$, 90% CI $[.130, .248]$, $\text{BF}_{\text {10}} = 2.28 \times 10^{34}$), supporting hypothesis H1b. Specifically, the median RT was higher for the more difficult level in every contrast, with two exceptions: It did not differ between 2- and 4-back, and it was higher for 3- than for 4-back (Table 1).

**Table 1:** Paired contrasts for the rmANOVA comparing the median reaction time between *n*-back levels.

Contrast	Estimate	*SE*	*df*	*t*	*p*	$\text{BF}_{\text {10}}$	$\eta _{p}^{2}$	$95\% \,CI$
1–2	$-$0.11	0.01	345.00	$-$11.76	<0.001	$1.75 \times 10^{30}$	0.29	[0.22, 1.00]
1–3	$-$0.16	0.01	345.00	$-$16.23	<0.001	$8.80 \times 10^{45}$	0.43	[0.37, 1.00]
1–4	$-$0.12	0.01	345.00	$-$12.47	<0.001	$4.79 \times 10^{34}$	0.31	[0.25, 1.00]
2–3	$-$0.04	0.01	345.00	$-$4.47	<0.001	5538.45	0.05	[0.02, 1.00]
2–4	$-$0.01	0.01	345.00	$-$0.71	0.894	0.10	1.45e−03	[0.00, 1.00]
3–4	0.04	0.01	345.00	3.76	0.001	$6.35 \times 10^{6}$	0.04	[0.01, 1.00]

The column Contrast contains the *n* of the *n*-back levels. *SE* = standard error, *df* = degrees of freedom, *t* = *t*-statistic, *p* = *p*-value, *CI* = confidence interval.

All NASA-TLX subscale scores increased across $n$-back levels, so evidence was in favour of H1c. Ratings on the effort subscale ($F(2.20, 253.06) = 203.82$, $p < 0.001$, $\hat{\eta }^2_G = 0.316$, 90% CI $[0.250, 0.375]$, $\text{BF}_{\text {10}} = 2.47 \times 10^{34}$) increased across all levels, but the magnitude of change decreased from 1- to 2-back ($t(345) = -12.35$, $p_\mathrm {Tukey(4)} < 0.001$, $\text{BF}_{\text {10}} = 4.24 \times 10^{19}$) to 3- to 4-back ($t(345) = -2.72$, $p_\mathrm {Tukey(4)} = 0.035$, $\text{BF}_{\text {10}} = 174.38$). Three subscales had significant differences between all contrasts except for 3- versus 4-back: While ratings on the frustration and time subscales were higher for more difficult levels ($F(2.50, 287.66) = 68.06$, $p < 0.001$, $\hat{\eta }^2_G = 0.172$, 90% CI $[0.112, 0.227]$, $\text{BF}_{\text {10}} = 5.26 \times 10^{15}$, and $F(2.21, 254.65) = 51.08$, $p < 0.001$, $\hat{\eta }^2_G = 0.117$, 90% CI $[0.065, 0.168]$, $\text{BF}_{\text {10}} = 3.94 \times 10^{9}$, respectively), ratings on the performance subscale decreased with higher $n$ ($F(2.49, 285.97) = 95.33$, $p < 0.001$, $\hat{\eta }^2_G = 0.241$, 90% CI $[0.176, 0.299]$, $\text{BF}_{\text {10}} = 1.55 \times 10^{24}$). Ratings on the mental subscale consistently increased across all levels ($F(1.99, 228.35) = 274.47$, $p < 0.001$, $\hat{\eta }^2_G = 0.375$, 90% CI $[0.309, 0.432]$, $\text{BF}_{\text {10}} = 1.64 \times 10^{43}$). Ratings on the physical subscale were higher for more difficult levels ($F(1.68, 192.93) = 15.91$, $p < 0.001$, $\hat{\eta }^2_G = 0.041$, 90% CI $[0.009, 0.075]$, $\text{BF}_{\text {10}} = 60.54$), apart from the contrasts 2- versus 3-back ($\text{BF}_{\text {10}} = 10.45$) and 3- versus 4-back ($\text{BF}_{\text {10}} = 0.47$). The full results of these manipulation checks are listed in Tables S.1–S.8 in the Supplementary Material.

### Decline of subjective values

**Figure 2:**
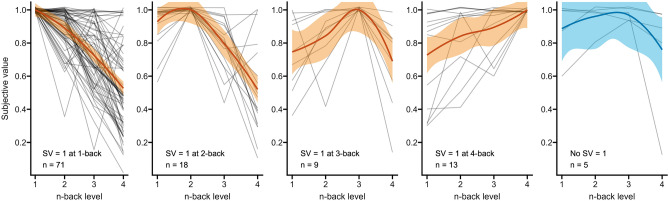
Subjective values (SV) per $n$-back level, grouped into those who had an SV = 1 for 1-back, for 2-back, for 3-back, for 4-back, or no SV = 1 for any level. The lines have a vertical jitter of 0.02. Smoothing of conditional means with Loess method. Transparent overlays depict the 95% confidence interval. Figure available at osf.io/vnj8x/, under a CC-BY-4.0 license.

The different curves of SVs across $n$-back levels can be seen in Fig. 2, grouped into those participants who had an SV of 1.0 for 1-back ($n=71$), for 2-back ($n=18$), for 3-back ($n=9$), for 4-back ($n=13$), or all SVs below 1.0, i.e. no absolute preference for any level ($n=5$). While the majority of participants preferred the easiest level and showed an approximately linear decline of SVs with increasing task-load, a substantial part of the sample had higher SVs for one of the more difficult $n$-back levels. However, each panel in Figure 2 contains curves of participants who had large differences between their four SVs and curves of participants who had a difference of less than 0.2 between their highest and their lowest SV, so preferring one level does not necessarily mean having a strong aversion against the others, regardless of difficulty level.

**Figure 3:**
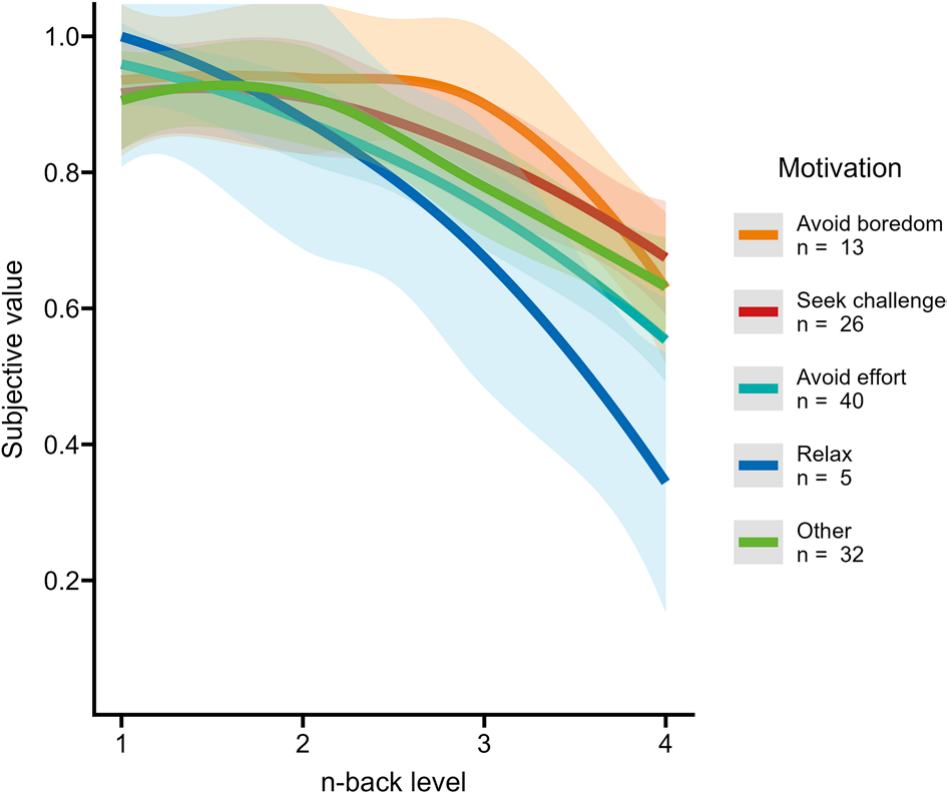
Trajectories of subjective values per $n$-back level for each participant, grouped by the motivation for effort discounting that they indicated in the single choice question after the paradigm. $N$ = 116. 'Other' opened up a free text field. Smoothing of conditional means with Loess method. Transparent overlays depict the 95% confidence interval. Figure available at osf.io/vnj8x/, under a CC-BY-4.0 license.

When asking participants what motivated their decisions in the cognitive effort discounting paradigm, 11.2% stated that they wanted to avoid boredom, 22.4% stated that they wanted a challenge, 34.5% stated that they wanted to avoid effort, and 4.3% stated that they wanted to relax. The remaining 27.6% of participants used the free text field and provided reasons such as “I wanted a fair relation of effort and reward”, “I wanted the fun that I had in the more challenging levels”, “I wanted to maximize reward first and minimize effort second”, or “I did not want to perform poorly when I was being paid for it”. Figure 3 shows the different motivations in the context of the SVs per $n$-back level.

The rmANOVA showed a significant difference between the SVs across $n$-back levels ($F(1.98, 227.98) = 65.65$, $p < 0.001$, $\hat{\eta }^2_G = 0.288$, 90% CI $[0.222, 0.347]$, $\text{BF}_{\text {10}} = 1.58 \times 10^{64}$), so evidence was in favour of H2a. All four pre-defined contrasts reached significance (Table 2), so a purely linear contrast can be rejected.

**Table 2:** Contrasts for the rmANOVA comparing the subjective values between *n*-back levels.

Contrast	Estimate	*SE*	*df*	*t*	*p*	$\eta _{p}^{2}$	$95\% \,CI$
Declining linear	1.11	0.08	345.00	13.41	<0.001	0.34	[0.28, 1.00]
Ascending quadratic	0.15	0.04	345.00	4.14	<0.001	0.05	[0.02, 1.00]
Declining logistic	1.22	0.09	345.00	12.97	<0.001	0.33	[0.26, 1.00]
Positively skewed normal	0.75	0.06	345.00	12.74	<0.001	0.32	[0.26, 1.00]

*SE* = standard error, *df* = degrees of freedom, *t* = *t*-statistic, *p* = *p*-value, *CI* = confidence interval.

The declining logistic contrast had the highest effect estimate ($t(345) = 12.97$, $p < 0.001$), suggesting a shallow decline of SVs between 1- and 2-back, and 3- and 4-back, respectively, and a steeper decline of SVs between 2- and 3-back. Based on the effect estimate, the ascending quadratic and the skewed normal contrasts were rejected in favour of the declining logistic contrast.

Consequently, we had to adapt the MLM to incorporate this non-linear trend. To apply the contrast to the $n$-back levels, we had to turn the variables into a factor, with two consequences: Centered variables cannot be turned into factors, so we entered the variable level in its raw form, and factors cannot be used as random slopes, so the model is now defined as:

$$\begin{aligned} SV \sim level\ + d' + median \,RT + (1|subject) \end{aligned}$$

This means that the intercept still varied between subjects, but there were no random slopes anymore. To provide more than one observation per factor level, we used the two rounds per $n$-back level per subject, rather than $n$-back levels per subject. The ICC of the null model indicated that there was a correlation of $r=$ 0.096 between the SVs of a subject, i.e. that 9.59% of variance in SVs could be explained by differences between participants. We did not use an optimization algorithm to improve the fit of the random intercept model. A total of 9 data points from 6 participants were excluded, because the residuals exceeded 3 SD above the mean. The results of the final model are displayed in Table 3.

**Table 3:** Results of the multi level model on the influence of *n*-back level (as a declining logistic contrast) and task performance on subjective values.

Parameter	Beta	*SE*	*df*	*t*-value	*p*-value	$f^{2}$	Random Effects (SD)
Intercept	0.81	0.01	115.00	78.68	<.001		0.09
n-back level	0.03	0.00	797.54	9.99	<.001	0.21	
d′	0.21	0.03	797.63	6.28	<.001	0.05	
Median RT	0.03	0.07	797.80	0.42	0.674	0.00	

*SE* = standard error, *df* = degrees of freedom, *SD* = standard deviation.

An exploratory ANOVA was used to compare the fit of the final model with a linear random intercept model, confirming that the two models were different from each other ($\chi ^2 (2)$ = 28.35, $p<0.001$), and with an Akaike Information Criterion of $AIC=-510.93$ and a Bayesian Information Criterion of $BIC=-472.35$ the declining logistic model was superior to the linear model ($AIC=-486.58$, $BIC=-457.65$). Both AIC and BIC subtract the likelihood of the model from the number of parameters and/or data points, so lower values indicate better model fit. The final model had an effect size of $f^{2}=$ 0.21 for the $n$-back levels and $f^{2}=$ 0.05 for $d'$, which are considered medium and small, respectively^46^. This means that the $n$-back level explained 20.67% and $d'$ explained 4.90% of variance in SVs relative to the unexplained variance, respectively. The beta coefficient indicated that with every 1-unit increase in $d'$, the SV increased by 0.21. Due to the coding scheme of the logistic contrast, the beta coefficient of the $n$-back level has to be interpreted inversely, so SVs decline with increasing $n$-back level. The effect size of the median RT was $f^{2}=$ 0.00. Since SVs decline with increasing level, beyond the variance explained by $d'$, evidence was in favour of H2b.

**Figure 4:**
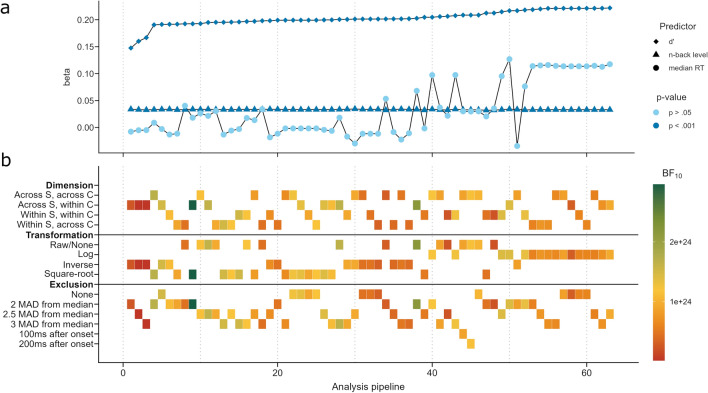
Results of the multi level model for each of the 63 preprocessing pipelines. Drawing a vertical through both panels indicates the type of preprocessing (panel b) of the pipeline and the resulting beta estimates of the three predictors in the model (panel a). The colourbar in panel b indicates the BF10 of each multi level model compared to a model in which the $n$-back level has no effect. The pipelines in both panels are sorted left to right in ascending order of the magnitude of the beta estimate of the predictor $d'$. Figure available at osf.io/vnj8x/, under a CC-BY-4.0 license.

To investigate the dependency of the model results on the RT preprocessing, we conducted a specification curve analysis (Figure 4).

Regardless of the preprocessing pipeline, $n$-back level and $d'$ were significant predictors of SVs, and had stable effect estimates across all pipelines. There was no pipeline in which the median RT was a significant predictor of SVs, but it showed volatile effect estimates across pipelines. The pipelines that yielded the highest estimates for $d'$ and the median RT used log-transformed data, irrespective of the dimension and exclusion criteria.

### Differences between NFC groups

The median NFC was 16, with $n=57$ subjects below and $n=59$ above the median. We used an rmANOVA to investigate whether the difference between the SVs of 1- and 2-back, and 2- and 3-back, respectively, depended on whether a participant’s NFC score was above or below the median. There was a main effect of the $n$-back level ($F(1, 114) = 9.13$, $p = 0.003$, $\hat{\eta }^2_G = 0.040$, 90% CI $[.002, .115]$, $\text{BF}_{\text {10}} = 12.68$), but neither a main effect of the NFC group ($F(1, 114) = 3.18$, $p = 0.077$, $\hat{\eta }^2_G = 0.013$, 90% CI $[0.000, 0.068]$, $\text{BF}_{\text {10}} = 0.56$) nor an interaction of NFC group and $n$-back level ($F(1, 114) = 0.46$, $p = 0.499$, $\hat{\eta }^2_G = 0.002$, 90% CI $[0.000, 0.037]$), so evidence was not in favour of H3a. Post-hoc tests showed that the difference between the SVs of 2- and 3-back is slightly more negative than the difference between 1- and 2-back ($t(114) = -3.02$, $p = 0.003$), but there were large inter-individual differences (Supplementary Fig. S2a). This means that across the whole sample, there was a steeper decline in SVs from 2- to 3-back than from 1- to 2-back, again resembling the declining logistic function.

The rmANOVA on the association between NFC scores and NASA-TLX scores revealed a main effect of $n$-back level ($F(2.10, 239.56) = 154.50$, $p < 0.001$, $\hat{\eta }^2_G = 0.223$, 90% CI $[0.159, 0.282]$, $\text{BF}_{\text {10}} = 2.22 \times 10^{45}$) and an interaction between $n$-back level and NFC scores ($F(2.10, 239.56) = 4.93$, $p = 0.007$, $\hat{\eta }^2_G = 0.009$, 90% CI $[0.000, 0.025]$), but no main effect of NFC scores ($F(1, 114) = 3.22$, $p = 0.075$, $\hat{\eta }^2_G = 0.022$, 90% CI $[0.000, 0.084]$, $\text{BF}_{\text {10}} = 1.75 \times 10^{2}$). Post-hoc tests showed that the participants with NFC scores below the median had higher NASA-TLX scores for 3-back ($t(114) = -2.15$, $p = 0.033$, $\text{BF}_{\text {10}} = 11.15$) and for 4-back ($t(114) = -2.89$, $p = 0.005$, $\text{BF}_{\text {10}} = 336.88$) than those with NFC scores above the median, so evidence was in favour of H3b. Regardless of NFC scores, NASA-TLX scores were higher for the more difficult level in each pair of $n$-back levels (Supplementary Fig. S3).

With another rmANOVA we investigated whether the difference between the aversiveness scores of 1- and 2-back, and 2- and 3-back, respectively, depended on whether a participant’s NFC score was above or below the median. There was a main effect of NFC group ($F(1, 114) = 8.43$, $p = 0.004$, $\hat{\eta }^2_G =0.043$, 90% CI $[0.003, 0.119]$, $\text{BF}_{\text {10}} = 14.26$) and a main effect of the $n$-back level ($F(1, 114) = 10.21$, $p = 0.002$, $\hat{\eta }^2_G = 0.034$, 90% CI $[0.000, 0.105]$, ), but no interaction ($F(1, 114) = 2.59$, $p = 0.110$, $\hat{\eta }^2_G = 0.009$, 90% CI $[0.000, 0.058]$). In favour of H3c, post-hoc tests revealed that participants with NFC scores below the median reported higher aversiveness than participants with NFC scores above the median ($t(114) = 2.90$, $p = 0.004$) (Supplementary Fig. S2b). Regardless of NFC, the difference of the aversiveness scores of 2- and 3-back was more negative than that of 1- and 2-back ($t(114) = 3.20$, $p = 0.002$), indicating that in the same way in which the SVs decreased more strongly from 2- to 3-back than from 1- to 2-back, the aversion increased more strongly. The full results of these analyses of NFC group differences can be found in Tables S.11–S.15 in the Supplementary Material.

## Exploratory analyses

To investigate the apparent group difference between the SVs of participants with NFC scores below and above the median in higher $n$-back levels, we computed an rmANOVA with the within-factor level (1 to 4) and the between-factor NFC group (below/above median). There was no main effect of NFC group ($F(1, 114) = 2.63$, $p = 0.108$, $\hat{\eta }^2_G = 0.007$, 90% CI $[0.000, 0.053]$, $2.95 \times 10^{-1}$), but a main effect of the $n$-back level ($F(2.01, 229.39) = 67.39$, $p < 0.001$, $\hat{\eta }^2_G = 0.295$, 90% CI $[0.228, 0.354]$, $2.70 \times 10^{30}$) and an interaction ($F(2.01, 229.39) = 3.24$, $p = 0.041$, $\hat{\eta }^2_G = 0.020$, 90% CI $[0.000, 0.044]$). Post-hoc tests for the main effect of level showed that SVs were lower for the more difficult $n$-back level in each paired contrast except for 1- versus 2-back. Post-hoc tests for the interaction effect showed that the NFC groups only had a significant difference in SVs for 4-back, where participants below the NFC median had lower scores ($\Delta M = 0.11$, 95% CI $[0.01, 0.22]$, $t(114) = 2.13$, $p = 0.036$). Despite not reaching significance, 1-back was the only level in which participants with NFC scores above the median seemed to have lower SVs than those with scores below the median ($\Delta M = -0.05$, 95% CI $[-0.11, 0.01]$, $t(114) = -1.50$, $p = .136$). The full results of this exploratory analysis of NFC group differences can be found in Tables S.16 and S.17 in the Supplementary Material. Supplementary Fig. S4 shows the SVs per $n$-back level for participants with NFC scores above and below the median.

**Figure 5:**
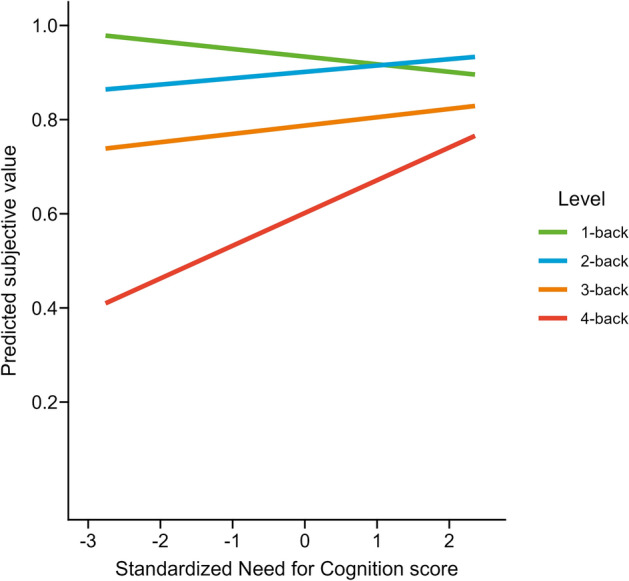
Predicted slopes of subjective values depending on individual Need for Cognition scores for each $n$-back level. The slopes of 1-back and 4-back are different at $p$ = .01. $N$ = 116. Figure available at osf.io/vnj8x/, under a CC-BY-4.0 license.

Following a reviewer’s recommendation, we also analyzed the association of SVs with NFC as a continuous variable. We computed an rmANOVA with the $n$-back level as a within variable and the standardized NFC score as a covariate to predict SVs. Both the NFC score ($F(1, 114) = 4.34$, $p = 0.039$, $\hat{\eta }^2_G = 0.011$, 90% CI $[0.000, 0.063]$, $\text{BF}_{\text {10}} = 0.57$) and the $n$-back level ($F(2.02, 229.75) = 67.24$, $p < 0.001$, $\hat{\eta }^2_G = 0.295$, 90% CI $[0.228, 0.354]$, $\text{BF}_{\text {10}} = 2.70 \times 10^{30}$) showed significant main effects, as well as a significant interaction ($F(2.02, 229.75) = 3.78$, $p = 0.024$, $\hat{\eta }^2_G = 0.023$, 90% CI $[0.000, 0.049]$, $\text{BF}_{\text {10}} = 0.12$). Analyzing the estimated marginal means of the linear trends for each $n$-back level indicated a significant difference between the slopes of 1-back and 4-back ($\Delta M = -0.09$, $95\%\ \text{CI}_\mathrm {Tukey(4)}$ $[-0.15, -0.02]$, $t(456) = -3.22$, $p_\mathrm {Tukey(4)} = 0.008$), but not between any other two levels. Plotting the predicted slopes shows that there is a negative association between the predicted SVs and the NFC scores for 1-back, but a positive association between the predicted SVs and the NFC scores for 4-back (Fig. 5). The full results of this exploratory analysis of NFC as a continuous covariate can be found in Tables S.18 and S.19 in the Supplementary Material.

## Discussion

This Registered Report aimed to adapt the Cognitive Effort Discounting (COG-ED) paradigm by Westbrook et al.^7^, which estimates subjective values of different $n$-back levels, into the Cognitive and Affective Discounting (CAD) paradigm to estimate SVs of tasks without defaulting to the assumed objective task load as a benchmark. For this purpose, we adapted the way in which the discounting options are presented to the participants, based the anchor on their own choices, and computed SVs across multiple combinations of task levels. The analyses were closely aligned with those in Westbrook et al.^7^ to demonstrate the changes in SVs brought about by the new paradigm. This study also applied the CAD paradigm to an emotion regulation task, the results of which are detailed in a second Registered Report^17^.

### Manipulation checks

Both $d'$ and the median RT changed across $n$-back levels, indicating that there was an increase in objective task load. The steepest decrease in $d'$ between levels was from 2- to 3-back, resembling the declining logistic curve of the SVs. Interestingly, the median RT increased from 2- to 3-back, but did not differ between 2- and 4-back. Since feedback was given after each round, this pattern could be interpreted in such a way that participants tried to compensate their lower accuracy in 3-back by relying more on luck than on memory in 4-back and prioritized speed over accuracy. Furthermore, several participants said that they perceived 3-back as more difficult than 4-back because they found it is easier to remember chunks of stimuli when $n$ was an even number than when $n$ was an odd number. This would support the notion that the manipulation of task load in an $n$-back task is not strictly continuous. And lastly, the fact that neither accuracy nor speed is an informative performance measure by itself has been observed before^47^ and both show different associations with various measures of intelligence^4^, suggesting that they should always be reported as separate indices.

All NASA-TLX subscales differed across $n$-back levels, but the effort and mental load subscales were the only ones to consistently increase across all levels. This would support the notion of the $n$-back task offering a continuous manipulation of task load, at least subjectively. Ratings on the frustration and time subscales increased and ratings on the performance subscale decreased until 3-back and then remained stable. This pattern is akin to the RT, which also increased and then remained stable. Ratings on the physical load subscale increased with $n$-back levels, but not between 2- and 3-back and 3- and 4-back, respectively.

### Decline of subjective values

The rmANOVA with different pre-defined contrasts showed that all fit the SVs to a different degree, and that the SVs do not simply decline linearly across $n$-back levels. The best fit was a declining logistic curve, reflecting that (1) the majority of participants preferred the easiest level, 2) 2-back was generally closer in preference to 1-back than 3-back was to 2-back, and (3) objective task load and subjective preference do not stand in a linear relationship. Since the majority of participants preferred the easiest level, we rejected the ascending quadratic and skewed normal contrasts, which implied lower SVs for 1- than for 2-back. The fact that the majority of participants preferred lower over higher effort, but a minority showed the opposite pattern, is in line with previous research on cognitive effort by Kool et al.^48^. Importantly, having a paradigm that can accurately assess the preferences of the minority is necessary but not sufficient, because the interindividual variability is so high that it blurs effects on the group level. Figure 2 suggests that those who prefer either 1- or 2-back have a slightly steeper discounting curve than those who prefer 3- or 4-back, meaning they have lower SVs for higher levels than those who prefer a higher level have for the easier levels. But as the figure also shows, there is great interindividual variability in the discounting patterns, regardless of which level has the highest SV. Thomson and Oppenheimer^49^ argue that the different effort curves that have been observed for different tasks are likely due to the fact that we still understand quite little about how and why different manipulations of effort work. For example, the $n$-back task is likely not a continuous manipulation of task load, as discussed above. However, the declining logistic curve is similar to the sigmoidal curve that has been found for a physical^50^ and a cognitive effort paradigm^51^, suggesting there are common features of effort across different tasks and domains. The MLM with the logistic contrast showed that the $n$-back level explained the majority of variance in SVs, while the performance measure $d'$ also explained some variance in SVs, albeit less. With increasing $n$-back level and decreasing $d'$, the SV decreased. The median RT was not a significant predictor in this model, which was somewhat surprising because RT but not $d'$ yielded significant differences across levels in the manipulation checks. However, participants might have deliberately or subconsciously used the feedback they received at the end of each round, i.e. twice per $n$-back level, as an anchor during the effort discounting. This feedback was based on correct responses and not on RT, so if participants based their effort discounting choices at least partly on this feedback, they were either motivated to repeat a task in which they performed well and/or they were reluctant to accept a larger reward for a task in which they did not perform well. Since more participants reported effort avoidance as their motivation in the effort discounting than those who reported seeking a challenge, we can assume that they were more motivated to repeat a task in which they performed well because their good performance coincided with low effort.

The declining logistic $n$-back levels and $d'$ remained significant predictors of SVs throughout all 63 preprocessing pipelines in the specification curve analysis. The effect estimates of the $n$-back level varied by about 0.002, those of $d'$ by about 0.074. In contrast to this stood the variability of the median RT betas (around 0.16), which did not reach significance in any pipeline. Interestingly, the curve of median RT betas in Fig. 4a mirrored the rectangular pipeline indicators in the transformation rows of Fig. 4b, so the transformation choice influenced the median RT much more than the dimension or the exclusion choice did. As Fernandez et al.^52^ found, applying more than one preprocessing step to the reaction time data of a Stroop task increased the risk of false positives beyond $\alpha =0.05$, and transformation choices inflated this risk more than outlier exclusion or aggregation choices did. Our data seems to corroborate this finding for $n$-back tasks as well. Surprisingly, the $d'$ betas appear almost unaffected by the preprocessing pipeline, even though $d'$ was computed after the outlier exclusion. This indicates that researchers who are interested in the correctness rather than the speed of responses can choose a simple preprocessing pipeline without risking false positives through elaborate transformations.

### Differences between NFC groups

The majority of participants (61.20 %) had a preference for 1-back over the other levels, but that also means that there were 34.50 % who had a preference for 2-, 3-, or 4-back, and 4.30 % who preferred no specific level over all others. It shows that when given the choice, there is a number of participants who do not prefer the easiest level, confirming the necessity of an effort discounting paradigm that works independent of the objective task load. The CAD paradigm provides the means to depict these preferences.

In the analysis of SV difference scores, the NFC group did not reach significance as a predictor. Conceptually, this was likely due to the partial but not full overlap between self-reported and behavioural effort investment, which has also been found for a demand selection task^53^. Another possible reason is the bandwidth of SVs of participants with NFC scores around the median, and the fact that the difference appeared most pronounced for 4-back, and we only registered analyses of the difference scores between 1- and 2-back and 2- and 3-back. As the exploratory analyses showed, a median split of NFC scores yielded a significant group difference in SVs for 4-back only, while predicting SVs with NFC as a continuous covariate showed a difference in the slopes of 1-back and 4-back. The analysis of NASA-TLX scores showed that the sum score increased with every $n$-back level, and that participants with NFC scores below the median had higher NASA-TLX scores for 3- and 4-back than those below the median. This demonstrates that higher $n$-back levels have a higher discriminatory power regarding inter-individual differences in subjective effort perception. This was also supported by the fact that higher $n$-back levels were perceived as more aversive, and participants with NFC scores below the median reported higher aversion than those with NFC scores above the median. Our data supports the notion of a Nonlinear Interaction between Person and Situation that has also been described by Schmitt et al.^54^ and Blum et al.^55^ in the same-named NIPS model. The NIPS model describes behaviour as a function of situational affordance which is mediated by personality traits. The behavioural variability follows an s-shaped curve, such that “strong” situations with low or high situational affordance elicit the least behavioural variability, while “weak” situations with moderate affordance maximize individual differences. These differences are caused by a person’s expression of a certain trait, which shifts the curve along the y-axis. In our study, the situational affordance is the $n$-back level and the behaviour is the SV, following a declining logistic curve, i.e. a mirrored s-shape. Hence, the variability in SVs increased from 1- to 4-back, and participants with higher NFC showed a more shallow decline in SVs as the situational affordance approached moderate values. According to the NIPS model, we can expect the SVs of participants with higher and lower NFC to converge again in levels of $n>4$, since behavioural variability decreases when situational affordance is high. An investigation of this relationship using the COG-ED paradigm^7^ had been encouraged by Strobel et al.^53^ based on their findings on demand avoidance and cognitive effort investment. With the CAD paradigm, the declining logistic contrast of SVs across levels resembles the ascending logistic curve of the NIPS model^54,55^ and should be tested further in a setting with $n$-back levels exceeding $n=4$.

### Limitations

When developing a new paradigm, it is challenging to decide on the optimal analysis strategy, as every hypothesis is based on expected data patterns rather than previous findings. While the Stage 1 review process made the analyses as robust as possible, there were still unknown factors that should be addressed by future studies. For instance, the differences between participants with higher and lower NFC should be investigated with extreme groups or as a continuous variable rather than with a median split, especially in academic samples where NFC can be expected to be higher on average and more narrow in range. To arrive at a sample with more balanced NFC scores, recruitment efforts should be focused on representative population samples and/or collecting data with an NFC-based stop rule. Additionally, we expected the SVs of participants with lower NFC scores to peak at 1-back and the SVs of those with higher scores to peak at 2-back, but the way the SVs of both groups appeared to drift apart in the higher $n$-back levels suggests that an analysis of those levels would be more fruitful in determining group differences. Future studies could create a stronger separation between the concepts investigated in this study (discounting curve, effort perception, performance, SV computation, NFC), and model the SVs and their task-related influencing factors first, before looking at (non-linear) associations with personality. Another important point is the instruction, not just for the $n$-back task, but for the effort discounting as well. We had to exclude several participants for misunderstanding the task instruction, so we will add a visual instruction and/or a training next time. And even though the participants were instructed to do the effort discounting with the aim to be satisfied with their choices instead of trying to increase the rewards, we cannot be sure that they did so. One might also argue that the 2€ reward range was not large enough to be an incentive for effort expenditure. However, findings by Bialaszek et al.^56^ suggest that participants are actually more sensitive to effort when the reward is small. Nevertheless, we exceeded the largest required sample size by 2.2 times, which gives our analyses high statistical power.

## Conclusion

Effort and reward are relevant in everyday life, yet these constructs vary in their conceptualization across individuals and even studies. With each decision an individual makes, they must weigh the required effort against the expected reward to decide if and how to behave in that situation. So far, effort discounting paradigms have relied on the assumption that the task that is objectively easiest is the one that is preferred by everyone, and each more difficult task is simply being devalued compared to the easy one. However, effort-related traits such as Need for Cognition suggest that this is not the case. Therefore, we developed a paradigm that allows to examine effort discounting independent of objective task load, which we tested using an $n$-back task. Despite the fact that the task design allowed individuals to express a preference for higher over lower objective load levels, the overall subjective values took the shape of a declining logistic curve across $n$-back levels. The majority of participants showed a decline in subjective values at higher effort levels. A minority of participants deviated from this pattern and showed a clear preference for 2-, 3, or 4-back over 1-back. The CAD paradigm was able to depict the individual preference patterns for both those who do and do not prefer the lowest effort level. While the subjective values declined with increasing levels, they increased with better performance as measured in $d'$, and were unaffected by the reaction time. Participants with Need for Cognition scores above the median reported lower subjective task load in and less aversion to more difficult levels. However, they did not have higher subjective values per se, which was due to our choice of median split and our assumption that these group differences would emerge in lower levels. The exploratory analyses showed that the predicted slope of subjective values depending on Need for Cognition scores differed between 1- and 4-back, but not between other levels. In fact, the reaction time and self-report data suggest that individual differences emerge especially from 3-back upwards, emphasizing the need for tasks with high discriminatory power and effort discounting paradigms with flexible, participant-centered mechanisms. The CAD paradigm offers this flexibility, and we encourage future studies to question traditional assumptions in the field of effort discounting in the light of these findings, and to re-use this data set for exploratory analyses.

### Supplementary Information

Supplementary Information.

## Data Availability

The data of this study can be downloaded from 10.17605/osf.io/vnj8x.

## Code availability

The paradigm code, the R script for analysis, and the R Markdown file used to compile this document are available at 10.17605/osf.io/vnj8x.
