# Supplementary material for: Need for Cognition is associated with a preference for higher task load in effort discounting
Source: Sci Rep. 2023 Nov 9;13:19501. doi: 10.1038/s41598-023-44349-3 (PMC10636210; doi:10.1038/s41598-023-44349-3)
Supplement: Supplementary file 1 — Supplementary Information. [file 41598_2023_44349_MOESM1_ESM.pdf]

Supplementary Material for the Registered Report titled: ‘When easy is not preferred: A discounting paradigm to assess load-independent task preference’

Josephine Zerna[†,1], Christoph Scheffel[†,1], Corinna Kührt[1], & Alexander Strobel[1]

[1] Faculty of Psychology, Technische Universität Dresden, 01069 Dresden, Germany

Author Note

The authors made the following contributions. Josephine Zerna: Conceptualization, Data curation, Methodology, Funding acquisition, Formal analysis, Investigation, Project administration, Software, Visualization, Writing - original draft, Writing - review & editing; Christoph Scheffel: Conceptualization, Methodology, Funding acquisition, Investigation, Project administration, Software, Writing - review & editing; Corinna Kührt: Formal analysis, Writing - review & editing; Alexander Strobel: Conceptualization, Resources, Supervision, Funding acquistion, Writing - review & editing. [†] Josephine Zerna and Christoph Scheffel contributed equally to this work.

Correspondence concerning this article should be addressed to Josephine Zerna, Zellescher Weg 17, 01069 Dresden, Germany. E-mail: josephine.zerna@tu-dresden.de

Supplementary Material for the Registered Report titled: ‘When easy is not preferred: A discounting paradigm to assess load-independent task preference’

Design Table

(Starts on next page)

The effect sizes for each hypothesis were taken from the corresponding analysis in Westbrook et al. (2013). There are two exceptions due to the fact that the information in Westbrook et al. (2013) was insufficient in that case: Hypothesis 1c was based on Kramer et al. (2021), and hypothesis 3b was based on our pilot data.

**Question**	**Hypothesis**	**Sampling plan (e.g. power analysis)**	**Analysis Plan**	**Interpretation given to different outcomes**
1. Do objective and subjective measures of performance reflect an increase in task load with increasing n-back level?	1a) The signal detection measure d' declines with increasing n-back level.	F tests - ANOVA: Repeated measures, within factors<br>Analysis: A priori: Compute required sample size<br><u>Input</u>:<br>Effect size f = 0.8685540<br>α err prob = 0.05<br>Power (1-β err prob) = 0.95<br>Number of groups = 1<br>Number of measurements = 4<br>Corr among rep measures = 0.5<br>Nonsphericity correction ε = 1<br><u>Output</u>:<br>Noncentrality parameter λ = 30.1754420<br>Critical F = 3.4902948<br>Numerator df = 3.0000000<br>Denominator df = 12.0000000<br>Total sample size = 5<br>Actual power = 0.9824202	Repeated measures ANOVA with six linear contrasts, comparing the d' value of two n-back levels (1, 2, 3, 4) at a time.<br>The ANOVA is calculated using aov_ez() of the afex-package, estimated marginal means are calculated using emmeans() from the emmeans-package, and pairwise contrasts are calculated using pairs().<br>Bayes factors are computed for the ANOVA and each contrast using the BayesFactor-package.	ANOVA yields $p < .05$ is interpreted as d' changing significantly with n-back levels.<br>Each contrast yielding $p < .05$ is interpreted as d' being different between those levels, magnitude and direction are inferred from the respective estimate.<br>The Bayes factor *BF10* is reported alongside every *p*-value to assess the strength of evidence.
	1b) Reaction time increases with increasing n-back level.	F tests - ANOVA: Repeated measures, within factors<br>Analysis: A priori: Compute required sample size<br><u>Input</u>:<br>Effect size f = 0.2041241<br>α err prob = 0.05<br>Power (1-β err prob) = 0.95<br>Number of groups = 1<br>Number of measurements = 4	Repeated measures ANOVA with six linear contrasts, comparing the median reaction time of two n-back levels (1, 2, 3, 4) at a time.<br>The ANOVA is calculated using aov_ez() of the afex-package, estimated marginal means are calculated using emmeans()	ANOVA yields $p < .05$ is interpreted as the median reaction time changing significantly with n-back levels.<br>Each contrast yielding $p < .05$ is interpreted as the median reaction time being different between those levels, magnitude

		Corr among rep measures = 0.5<br>Nonsphericity correction ε = 1<br>Output:<br>Noncentrality parameter λ = 17.6666588<br>Critical F = 2.6625685<br>Numerator df = 3.0000000<br>Denominator df = 156<br>Total sample size = 53<br>Actual power = 0.9506921	from the emmeans-package, and pairwise contrasts are calculated using pairs().<br><br>Bayes factors are computed for the ANOVA and each contrast using the BayesFactor-package.	and direction are inferred from the respective estimate.<br><br>The Bayes factor *BF10* is reported alongside every *p*-value to assess the strength of evidence.
	1c) Ratings on all NTLX subscales increase with increasing n-back level.	From Kramer et al. (2021):<br><br>F tests - ANOVA: Repeated measures, within factors<br>Analysis: A priori: Compute required sample size<br>Input:<br>Effect size f = 0.7071068<br>α err prob = 0.05<br>Power (1-β err prob) = 0.95<br>Number of groups = 1<br>Number of measurements = 4<br>Corr among rep measures = 0.5<br>Nonsphericity correction ε = 1<br>Output:<br>Noncentrality parameter λ = 24.0000013<br>Critical F = 3.2873821<br>Numerator df = 3.0000000<br>Denominator df = 15.0000000<br>Total sample size = 6<br>Actual power = 0.9620526	A repeated measures ANOVA for each NASA-TLX subscale, with six linear contrasts comparing the subscale score of two n-back levels (1, 2, 3, 4) at a time.<br><br>The ANOVA is calculated using aov_ez() of the afex-package, estimated marginal means are calculated using emmeans() from the emmeans-package, and pairwise contrasts are calculated using pairs().<br><br>Bayes factors are computed for the ANOVA and each contrast using the BayesFactor-package.	ANOVA yields $p < .05$ is interpreted as the subscale score changing significantly with n-back levels.<br><br>Each contrast yielding $p < .05$ is interpreted as the subscale score being different between those levels, magnitude and direction are inferred from the respective estimate.<br><br>The Bayes factor *BF10* is reported alongside every *p*-value to assess the strength of evidence.

2. Is the effort required for higher n-back levels less attractive, regardless of how well a person performs?	2a) Subjective values decline with increasing n-back level.	F tests - ANOVA: Repeated measures, within factors<br>Analysis: A priori: Compute required sample size<br>Input:<br>Effect size f = 0.9229582<br>α err prob = 0.05<br>Power (1-β err prob) = 0.95<br>Number of groups = 1<br>Number of measurements = 4<br>Corr among rep measures = 0.5<br>Nonsphericity correction ε = 1<br>Output:<br>Noncentrality parameter λ = 27.2592588<br>Critical F = 3.8625484<br>Numerator df = 3.0000000<br>Denominator df = 9.0000000<br>Total sample size = 4<br>Actual power = 0.9506771	Repeated measures ANOVA with four contrasts (linear (3,1,-1,-3), quadratic (-1,1,1,-1), logistic (3,2,-2,-3), and skewed normal (1,2,-1,-2)), comparing the subjective values of all n-back levels.<br>The ANOVA is calculated using aov_ez() of the afex-package, estimated marginal means are calculated using emmeans() from the emmeans-package.<br>Bayes factors are computed for the ANOVA and each contrast using the BayesFactor-package.	ANOVA yields *p* < .05 is interpreted as subjective values changing significantly with n-back levels.<br>Each contrast yielding *p* < .05 is interpreted as subjective values being different between levels, magnitude and direction are inferred from the respective estimate.<br>The Bayes factor *BF10* is reported alongside every *p*-value to assess the strength of evidence.
	2b) Subjective values decline with increasing n-back level, even after controlling for declining task performance measured by signal detection d' and reaction time.	F tests - ANOVA: Repeated measures, within factors<br>Analysis: A priori: Compute required sample size<br>Input:<br>Effect size f = 0.9229582<br>α err prob = 0.05<br>Power (1-β err prob) = 0.95<br>Number of groups = 1<br>Number of measurements = 4<br>Corr among rep measures = 0.5<br>Nonsphericity correction ε = 1<br>Output:<br>Noncentrality parameter λ = 27.2592588<br>Critical F = 3.8625484<br>Numerator df = 3.0000000	Multilevel model of SVs with n-back load level as level-1-predictor controlling for d' and reaction time subject-specific intercepts and allowing random slopes for n-back level.<br>The null model and the random slopes model are calculated using lmer() of the lmerTest-package.<br>Bayes factors are computed for the MLM using the BayesFactor-package.	Fixed effects yielding p < .05 are interpreted as subjective values changing significantly with n-back levels.<br>The Bayes factor BF10 is reported alongside every p-value to assess the strength of evidence.

		Denominator df = 9.0000000<br>Total sample size = 4<br>Actual power = 0.9506771		
3. Is there a discrepancy between perceived task load and subjective value of effort depending on a person's Need for Cognition?	3a) Participants with higher NFC scores have higher subjective values for 2- and 3-back but lower subjective values for 1-back than participants with lower NFC scores.	F tests - ANOVA: Repeated measures, within-between interaction: A priori: Compute required sample size<br>Input:<br>Effect size f = 0.57<br>α err prob = 0.05<br>Power (1-β err prob) = 0.95<br>Number of groups = 2<br>Number of measurements = 4<br>Corr among rep measures = 0.5<br>Nonsphericity correction ε = 1<br>Output:<br>Noncentrality parameter λ = 25.99<br>Critical F = 23.01<br>Numerator df = 3<br>Denominator df = 24<br>Total sample size = 10	Difference scores of subjective values are computed between consecutive n-back levels, and the sample is divided by their NFC median, so an rmANOVA with the within-factor n-back level and the between-factor NFC group can be computed.<br>The ANOVA is calculated using aov_ez() of the afex-package, estimated marginal means are calculated using emmeans() from the emmeans-package.<br>Bayes factors are computed for the ANOVA using the BayesFactor-package.	Subjective values are interpreted as being lower for 1-back and higher for 2- and 3-back in participants with higher NFC if there is a main effect of the NFC group ($p < .05$) and if the contrasts reveal that pattern at $p < .05$.<br>The Bayes factor BF10 is reported alongside every *p*-value to assess the strength of evidence.
	3b) Participants with higher NFC scores have lower NASA-TLX scores in every n-back level than participants with lower NFC scores.	Westbrook et al. have only reported the p-value here, so we used the ANOVA results of our pilot study, which included NASA-TLX scores (per level and subject) and NFC scores. The F statistic was $F(1,12) = 7.57$, which is an effect size of $f = 0.7355$.<br><br>F tests - ANOVA: Repeated measures, within-between interaction: A priori: Compute required sample size<br>Input:<br>Effect size f = 0.7355	NASA-TLX sum scores are computed per level and subject, and the sample is divided by their NFC median, so an rmANOVA with the within-factor n-back level and the between-factor NFC group can be computed.<br>The ANOVA is calculated using aov_ez() of the afex-package, estimated marginal means are	NASA-TLX scores are interpreted as being lower for participants with higher NFC if there is a main effect of the NFC group ($p < .05$) and if the contrasts reveal that pattern at $p < .05$.<br>The Bayes factor BF10 is reported alongside every p-value to assess the strength of evidence.

		α err prob = 0.05<br>Power (1-β err prob) = 0.95<br>Number of groups = 2<br>Number of measurements = 4<br>Corr among rep measures = 0.5<br>Nonsphericity correction ε = 1<br>Output:<br>Noncentrality parameter λ = 25.97<br>Critical F = 3.49<br>Numerator df = 3<br>Denominator df = 12<br>Total sample size = 6	calculated using emmeans() from the emmeans-package.<br><br>Bayes factors are computed for each predictor using the BayesFactor-package.	
	3c) Participants with higher NFC scores have lower aversiveness ratings for 2- and 3-back but higher higher aversiveness ratings for 1-back than participants with lower NFC scores.	As we could not find any study reporting an association of NFC and aversiveness ratings, we assumed a medium to large association (r = 0.25, according to Gignac & Szodorai (2016), doi: 10.1016/j.paid.2016.06.069). We assume this, because NFC is a trait defined as a preference for effortful cognitive activities, thereby it should be negatively associated with aversion to a cognitively effortful task.<br><br>F tests - ANOVA: Repeated measures, within-between interaction: A priori: Compute required sample size<br>Input:<br>Effect size f = 0.2582<br>α err prob = 0.05<br>Power (1-β err prob) = 0.95<br>Number of groups = 2<br>Number of measurements = 4<br>Corr among rep measures = 0.5<br>Nonsphericity correction ε = 1	Difference scores of aversiveness ratings are computed between consecutive n-back levels, and the sample is divided by their NFC median, so an rmANOVA with the within-factor n-back level and the between-factor NFC group can be computed.<br><br>The ANOVA is calculated using aov_ez() of the afex-package, estimated marginal means are calculated using emmeans() from the emmeans-package.<br><br>Bayes factors are computed for the ANOVA using the BayesFactor-package.	Aversiveness ratings are interpreted as being higher for 1-back and lower for 2- and 3-back in participants with higher NFC if there is a main effect of the NFC group ($p < .05$) and if the contrasts reveal that pattern at $p < .05$.<br><br>The Bayes factor BF10 is reported alongside every *p*-value to assess the strength of evidence.

		Output:<br>Noncentrality parameter λ = 18.13<br>Critical F = 2.70<br>Numerator df = 3<br>Denominator df = 96<br>Total sample size = 34		

Contrasts tested for in the repeated measures ANOVA

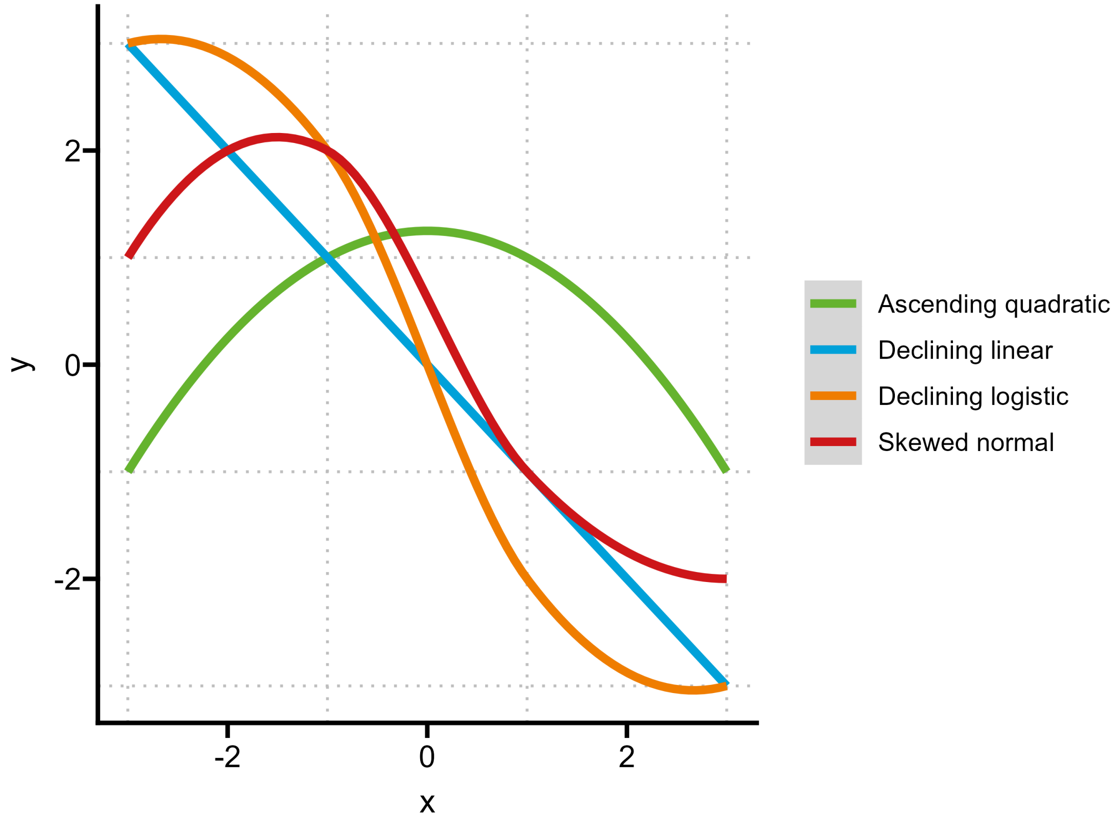

Figure S.1. Contrasts tested for in the repeated measures ANOVA that was used as a prerequisite for the multi level model of hypothesis 2b. Ascending quadratic (-1,1,1,-1), declining linear (3,1,-1,-3), declining logistic (3,2,-2,-3), and positively skewed normal (1,2,-1,-2). Figure available at osf.io/vnj8x/, under a CC-BY-4.0 license.

Hypothesis 1a: The signal detection measure d' declines with increasing n-back level.

ANOVA:

$F(2.74, 315.51) = 197.18$, $p < .001$, $\hat{\eta}^2_G = .464$, 90% CI [.403, .516], $\mathrm{BF}_{10} = 1.56 \times 10^{101}$

Paired contrasts:

Table S.1
Paired contrasts for the rmANOVA comparing d' between n-back levels.

Contrast	Estimate	SE	df	t	p	BF_{10}	η^2_p	$95\% CI$
1 - 2	0.09	0.02	345.00	4.31	0.00	93,954.04	0.05	[0.02, 1.00]
1 - 3	0.31	0.02	345.00	14.71	0.00	3.21×10^{47}	0.39	[0.32, 1.00]
1 - 4	0.46	0.02	345.00	21.89	0.00	4.46×10^{69}	0.58	[0.53, 1.00]
2 - 3	0.22	0.02	345.00	10.41	0.00	3.71×10^{23}	0.24	[0.18, 1.00]
2 - 4	0.37	0.02	345.00	17.58	0.00	2.01×10^{44}	0.47	[0.41, 1.00]
3 - 4	0.15	0.02	345.00	7.18	0.00	2.45×10^{14}	0.13	[0.08, 1.00]

Note. The column Contrast contains the n of the n-back levels. SE = standard error, df = degrees of freedom, t = t-statistic, p = p-value, CI = confidence interval.

Hypothesis 1b: The median reaction time increases with increasing n-back level.

ANOVA:

$F(2.46, 283.05) = 98.67$, $p < .001$, $\hat{\eta}^2_G = .192$, 90% CI $[.130, .248]$, $\mathrm{BF}_{10} = 2.28 \times 10^{34}$

Paired contrasts:

Table S.2

Paired contrasts for the rmANOVA comparing the median reaction time between n-back levels.

Contrast	Estimate	*SE*	*df*	*t*	*p*	BF_{10}	η^2_p	95%*CI*
1 - 2	-0.11	0.01	345.00	-11.76	<.001	1.75×10^{30}	0.29	[0.22, 1.00]
1 - 3	-0.16	0.01	345.00	-16.23	<.001	8.80×10^{45}	0.43	[0.37, 1.00]
1 - 4	-0.12	0.01	345.00	-12.47	<.001	4.79×10^{34}	0.31	[0.25, 1.00]
2 - 3	-0.04	0.01	345.00	-4.47	<.001	5,538.45	0.05	[0.02, 1.00]
2 - 4	-0.01	0.01	345.00	-0.71	0.894	0.10	1.45e-03	[0.00, 1.00]
3 - 4	0.04	0.01	345.00	3.76	0.001	6.35×10^{6}	0.04	[0.01, 1.00]

Note. The column Contrast contains the n of the n-back levels. SE = standard error, *df* = degrees of freedom, t = t-statistic, p = p-value, CI = confidence interval.

Hypothesis 1c: Ratings on all NASA-TLX dimensions increase with increasing n-back level.

Mental subscale ANOVA:

$F(1.99, 228.35) = 274.47$, $p < .001$, $\hat{\eta}^2_G = .375$, 90% CI $[.309, .432]$, $\mathrm{BF}_{10} = 1.64 \times 10^{43}$

Mental subscale paired contrasts:

Table S.3

Paired contrasts for the rmANOVA comparing ratings on the NASA-TLX Mental subscale between n-back levels.

Contrast	Estimate	SE	df	t	p	BF_{10}	η^2_p	95%CI
1 - 2	-3.91	0.34	345.00	-11.52	<.001	1.32×10^{23}	0.28	[0.21, 1.00]
1 - 3	-7.43	0.34	345.00	-21.91	<.001	1.83×10^{34}	0.58	[0.53, 1.00]
1 - 4	-8.91	0.34	345.00	-26.26	<.001	9.87×10^{36}	0.67	[0.62, 1.00]
2 - 3	-3.53	0.34	345.00	-10.40	<.001	1.09×10^{19}	0.24	[0.18, 1.00]
2 - 4	-5.00	0.34	345.00	-14.74	<.001	3.64×10^{22}	0.39	[0.32, 1.00]
3 - 4	-1.47	0.34	345.00	-4.35	<.001	3.83×10^{6}	0.05	[0.02, 1.00]

Note. The column Contrast contains the n of the n-back levels. SE = standard error, df = degrees of freedom, t = t-statistic, p = p-value, CI = confidence interval.

Physical subscale ANOVA:

$F(1.68, 192.93) = 15.91$, $p < .001$, $\hat{\eta}^2_G = .041$, 90% CI $[.009, .075]$, $\mathrm{BF}_{10} = 60.54$

Physical subscale paired contrasts:

Table S.4

Paired contrasts for the rmANOVA comparing ratings on the NASA-TLX Physical subscale between n-back levels.

Contrast	Estimate	*SE*	*df*	*t*	*p*	BF_{10}	η^2_p	95%*CI*
1 - 2	-0.95	0.32	345.00	-2.95	0.018	25.36	0.02	[0.00, 1.00]
1 - 3	-1.70	0.32	345.00	-5.29	<.001	602.59	0.08	[0.04, 1.00]
1 - 4	-2.04	0.32	345.00	-6.37	<.001	1,235.49	0.11	[0.06, 1.00]
2 - 3	-0.75	0.32	345.00	-2.34	0.092	10.45	0.02	[0.00, 1.00]
2 - 4	-1.09	0.32	345.00	-3.41	0.004	31.98	0.03	[0.01, 1.00]
3 - 4	-0.34	0.32	345.00	-1.07	0.705	0.47	3.33e-03	[0.00, 1.00]

Note. The column Contrast contains the n of the n-back levels. SE = standard error, df = degrees of freedom, t = t-statistic, p = p-value, CI = confidence interval.

Time subscale ANOVA:

$F(2.21, 254.65) = 51.08$, $p < .001$, $\hat{\eta}^2_G = .117$, 90% CI $[.065, .168]$, $\mathrm{BF}_{10} = 3.94 \times 10^9$

Time subscale paired contrasts:

Table S.5

Paired contrasts for the rmANOVA comparing ratings on the NASA-TLX Time subscale between n-back levels.

Contrast	Estimate	*SE*	*df*	*t*	*p*	BF_{10}	η^2_p	95%*CI*
1 - 2	-1.95	0.43	345.00	-4.53	<.001	7,366.95	0.06	[0.02, 1.00]
1 - 3	-4.28	0.43	345.00	-9.97	<.001	3.09×10^{10}	0.22	[0.16, 1.00]
1 - 4	-4.65	0.43	345.00	-10.81	<.001	2.02×10^{11}	0.25	[0.19, 1.00]
2 - 3	-2.34	0.43	345.00	-5.44	<.001	9.62×10^{6}	0.08	[0.04, 1.00]
2 - 4	-2.70	0.43	345.00	-6.28	<.001	8.02×10^{6}	0.10	[0.06, 1.00]
3 - 4	-0.36	0.43	345.00	-0.84	0.834	0.18	2.05e-03	[0.00, 1.00]

Note. The column Contrast contains the n of the n-back levels. SE = standard error, df = degrees of freedom, t = t-statistic, p = p-value, CI = confidence interval.

Performance subscale ANOVA:

$F(2.49, 285.97) = 95.33$, $p < .001$, $\hat{\eta}^2_G = .241$, 90% CI [.176, .299], $\mathrm{BF}_{10} = 1.55 \times 10^{24}$

Performance subscale paired contrasts:

Table S.6

Paired contrasts for the rmANOVA comparing ratings on the NASA-TLX Performance subscale between n-back levels.

Contrast	Estimate	*SE*	*df*	*t*	*p*	BF_{10}	η^2_p	95%*CI*
1 - 2	2.07	0.40	345.00	5.13	<.001	80,590.41	0.07	[0.03, 1.00]
1 - 3	5.14	0.40	345.00	12.74	<.001	1.23×10^{19}	0.32	[0.26, 1.00]
1 - 4	6.03	0.40	345.00	14.96	<.001	2.36×10^{19}	0.39	[0.33, 1.00]
2 - 3	3.07	0.40	345.00	7.61	<.001	8.72×10^{12}	0.14	[0.09, 1.00]
2 - 4	3.97	0.40	345.00	9.83	<.001	4.20×10^{12}	0.22	[0.16, 1.00]
3 - 4	0.90	0.40	345.00	2.22	0.119	2.35	0.01	[0.00, 1.00]

Note. The column Contrast contains the n of the n-back levels. SE = standard error, df = degrees of freedom, t = t-statistic, p = p-value, CI = confidence interval.

Effort subscale ANOVA:

$F(2.20, 253.06) = 203.82$, $p < .001$, $\hat{\eta}^2_G = .316$, 90% CI [.250, .375], $\mathrm{BF}_{10} = 2.47 \times 10^{34}$

Effort subscale paired contrasts:

Table S.7

Paired contrasts for the rmANOVA comparing ratings on the NASA-TLX Effort subscale between n-back levels.

Contrast	Estimate	*SE*	*df*	*t*	*p*	BF_{10}	η^2_p	95%*CI*
1 - 2	-4.23	0.34	345.00	-12.35	<.001	4.24×10^{19}	0.31	[0.24, 1.00]
1 - 3	-6.80	0.34	345.00	-19.84	<.001	4.25×10^{29}	0.53	[0.48, 1.00]
1 - 4	-7.73	0.34	345.00	-22.56	<.001	1.47×10^{32}	0.60	[0.55, 1.00]
2 - 3	-2.57	0.34	345.00	-7.49	<.001	3.85×10^{12}	0.14	[0.09, 1.00]
2 - 4	-3.50	0.34	345.00	-10.21	<.001	3.33×10^{15}	0.23	[0.17, 1.00]
3 - 4	-0.93	0.34	345.00	-2.72	0.035	174.38	0.02	[0.00, 1.00]

Note. The column Contrast contains the n of the n-back levels. SE = standard error, df = degrees of freedom, t = t-statistic, p = p-value, CI = confidence interval.

Frustration subscale ANOVA:

$F(2.50, 287.66) = 68.06$, $p < .001$, $\hat{\eta}^2_G = .172$, 90% CI $[.112, .227]$, $\mathrm{BF}_{10} = 5.26 \times 10^{15}$

Frustration subscale paired contrasts:

Table S.8

Paired contrasts for the rmANOVA comparing ratings on the NASA-TLX Frustration subscale between n-back levels.

Contrast	Estimate	*SE*	*df*	*t*	*p*	BF_{10}	η^2_p	95%*CI*
1 - 2	-2.17	0.43	345.00	-4.99	<.001	67,280.87	0.07	[0.03, 1.00]
1 - 3	-4.76	0.43	345.00	-10.94	<.001	8.22×10^{14}	0.26	[0.20, 1.00]
1 - 4	-5.57	0.43	345.00	-12.80	<.001	2.34×10^{15}	0.32	[0.26, 1.00]
2 - 3	-2.59	0.43	345.00	-5.95	<.001	6.41×10^{7}	0.09	[0.05, 1.00]
2 - 4	-3.40	0.43	345.00	-7.81	<.001	6.16×10^{8}	0.15	[0.10, 1.00]
3 - 4	-0.81	0.43	345.00	-1.86	0.246	0.92	9.96e-03	[0.00, 1.00]

Note. The column Contrast contains the n of the n-back levels. SE = standard error, df = degrees of freedom, t = t-statistic, p = p-value, CI = confidence interval.

Hypothesis 2a: Subjective values decline with increasing n-back level.

ANOVA:

$F(1.98, 227.98) = 65.65$, $p < .001$, $\hat{\eta}^2_G = .288$, 90% CI [.222, .347], $BF_{10} = 1.58 \times 10^{64}$

Pre-defined contrasts:

Table S.9
Contrasts for the rmANOVA comparing the subjective values between n-back levels.

Contrast	Estimate	*SE*	*df*	*t*	*p*	η^2_p	95%*CI*
Declining Linear	1.11	0.08	345.00	13.41	<.001	0.34	[0.28, 1.00]
Ascending Quadratic	0.15	0.04	345.00	4.14	<.001	0.05	[0.02, 1.00]
Declining Logistic	1.22	0.09	345.00	12.97	<.001	0.33	[0.26, 1.00]
Positively Skewed Normal	0.75	0.06	345.00	12.74	<.001	0.32	[0.26, 1.00]

Note. *SE* = standard error, *df* = degrees of freedom, *t* = *t*-statistic, *p* = *p*-value, CI = confidence interval.

Hypothesis 2b: Subjective values decline with increasing n-back level, even after controlling for declining task performance measured by signal detection d' and reaction time.

Multi level model:

Table S.10
Results of the multi level model on the influence of n-back level (as a declining logistic contrast) and task performance on subjective values.

Parameter	Beta	*SE*	*df*	*t*-value	*p*-value	f^2	Random Effects (SD)
Intercept	0.81	0.01	115.00	78.68	<.001		0.09
n-back level	0.03	0.00	797.54	9.99	<.001	0.21	
d'	0.21	0.03	797.63	6.28	<.001	0.05	
median RT	0.03	0.07	797.80	0.42	0.674	0.00	

Note. *SE* = standard error, *df* = degrees of freedom, *SD* = standard deviation.

The final model had an effect size of $f^2 = 0.21$ for the n-back levels and $f^2 = 0.05$ for d'. This means that the n-back level explained 20.67% and d' explained 4.90% of variance in SVs relative to the unexplained variance, respectively. The beta coefficient indicated that with every 1-unit increase in d', the SV increased by 0.21. The effect size of the median RT was $f^2 = 0.00$. The Bayes Factor of the full model against the null model was $BF_{10} = 7.83 \times 10^{29} \pm 16.28\%$.

Hypothesis 3a: Participants with higher NFC scores have higher subjective values for 2- and 3-back but lower subjective values for 1-back than participants with lower NFC scores.

ANOVA:

Main effect level: $F(1, 114) = 9.13$, $p = .003$, $\hat{\eta}^2_G = .040$, 90% CI $[.002, .115]$, $\mathrm{BF}_{10} = 12.68 \pm 0.00\%$

Main effect NFC group: $F(1, 114) = 3.18$, $p = .077$, $\hat{\eta}^2_G = .013$, 90% CI $[.000, .068]$, $\mathrm{BF}_{10} = 0.56 \pm 0.03\%$

Interaction: $F(1, 114) = 0.46$, $p = .499$, $\hat{\eta}^2_G = .002$, 90% CI $[.000, .037]$

Contrast for main effect level:

Table S.11
Paired contrast for the rmANOVA comparing the influence of Need for Cognition group and n-back level on difference scores of subjective values.

Contrast	Estimate	*SE*	*df*	*t*	*p*	BF_{10}	η^2_p	95%*CI*
1-2 - 2-3	-0.08	0.03	114.00	-3.02	0.003	12.68	0.07	[0.02, 1.00]

Note. The column Contrast contains the n of the n-back levels. SE = standard error, df = degrees of freedom, t = t-statistic, p = p-value, CI = confidence interval.

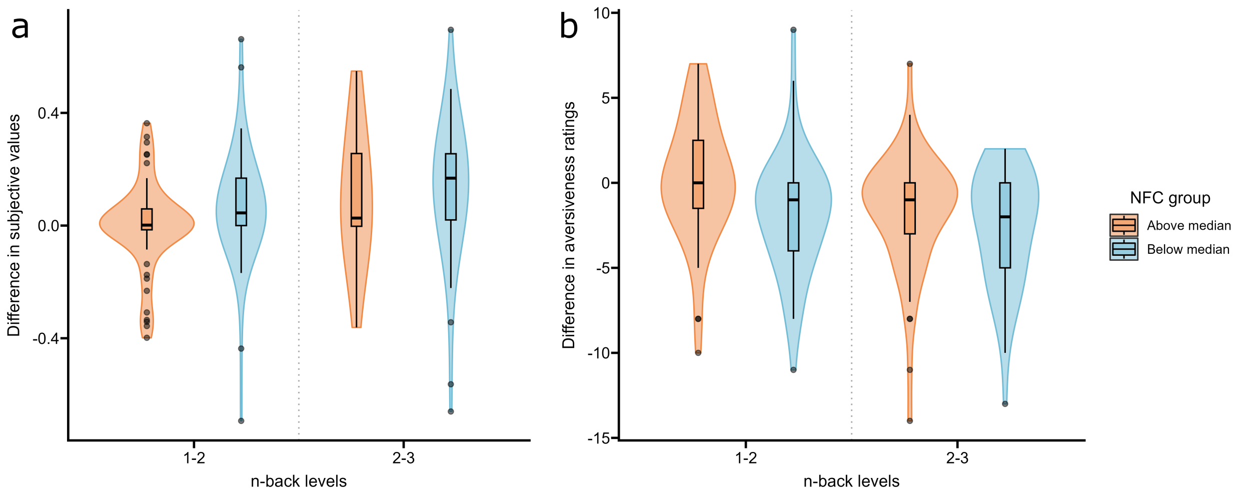

Figure S.2. Difference scores for subjective values (a) and aversiveness ratings (b) when subtracting 2- from 1-back and 3- from 2-back. Horizontal lines of the boxplots represent the median per group, whiskers represent 1.5 interquartile ranges. NFC = Need for Cognition score. $N = 116$. Figure available at osf.io/vnj8x/, under a CC-BY-4.0 license.

Hypothesis 3b: Participants with higher NFC scores have lower NASA-TLX scores in every n-back level than participants with lower NFC scores.

ANOVA:

Main effect level: $F(2.10, 239.56) = 154.50$, $p < .001$, $\hat{\eta}^2_G = .223$, 90% CI $[.159, .282]$, $\mathrm{BF}_{10} = 2.22 \times 10^{45}$

Main effect NFC group: $F(1, 114) = 3.22$, $p = .075$, $\hat{\eta}^2_G = .022$, 90% CI $[.000, .084]$, $\mathrm{BF}_{10} = 1.75 \times 10^{2}$

Interaction: $F(2.10, 239.56) = 4.93$, $p = .007$, $\hat{\eta}^2_G = .009$, 90% CI $[.000, .025]$

Contrast for main effect level:

Table S.12

Paired contrasts for the rmANOVA comparing the influence of Need for Cognition group and n-back level on NASA-TLX scores.

Contrast	Estimate	*SE*	*df*	*t*	*p*	BF_{10}	η^2_p	95%*CI*
1 - 2	-1.86	0.18	114.00	-10.06	<.001	7.02×10^{9}	0.47	[0.36, 1.00]
1 - 3	-3.31	0.23	114.00	-14.36	<.001	6.43×10^{29}	0.64	[0.56, 1.00]
1 - 4	-3.82	0.25	114.00	-15.32	<.001	5.25×10^{37}	0.67	[0.60, 1.00]
2 - 3	-1.46	0.16	114.00	-9.15	<.001	113,671.29	0.42	[0.31, 1.00]
2 - 4	-1.96	0.19	114.00	-10.39	<.001	4.18×10^{9}	0.49	[0.38, 1.00]
3 - 4	-0.51	0.13	114.00	-3.82	0.001	0.55	0.11	[0.04, 1.00]

Note. The column Contrast contains the n of the n-back levels. SE = standard error, df = degrees of freedom, t = t-statistic, p = p-value, CI = confidence interval.

Contrast for interaction:

Table S.13

Paired contrasts for the rmANOVA comparing the influence of Need for Cognition group and n-back level on NASA-TLX scores.

Contrast	Level	Estimate	SE	df	t	p	BF_{10}	η_p^2	$95\%CI$
high - low	1	-0.23	0.48	114.00	-0.48	0.632	0.18	2.02e-03	[0.00, 1.00]
high - low	2	-0.40	0.53	114.00	-0.76	0.450	0.25	5.00e-03	[0.00, 1.00]
high - low	3	-1.14	0.53	114.00	-2.15	0.033	11.15	0.04	[0.00, 1.00]
high - low	4	-1.53	0.53	114.00	-2.89	0.005	336.88	0.07	[0.01, 1.00]

Note. The column Contrast contains the groups above (high) and below (low) the Need for Cognition score median. SE = standard error, df = degrees of freedom, t = t-statistic, p = p-value, CI = confidence interval.

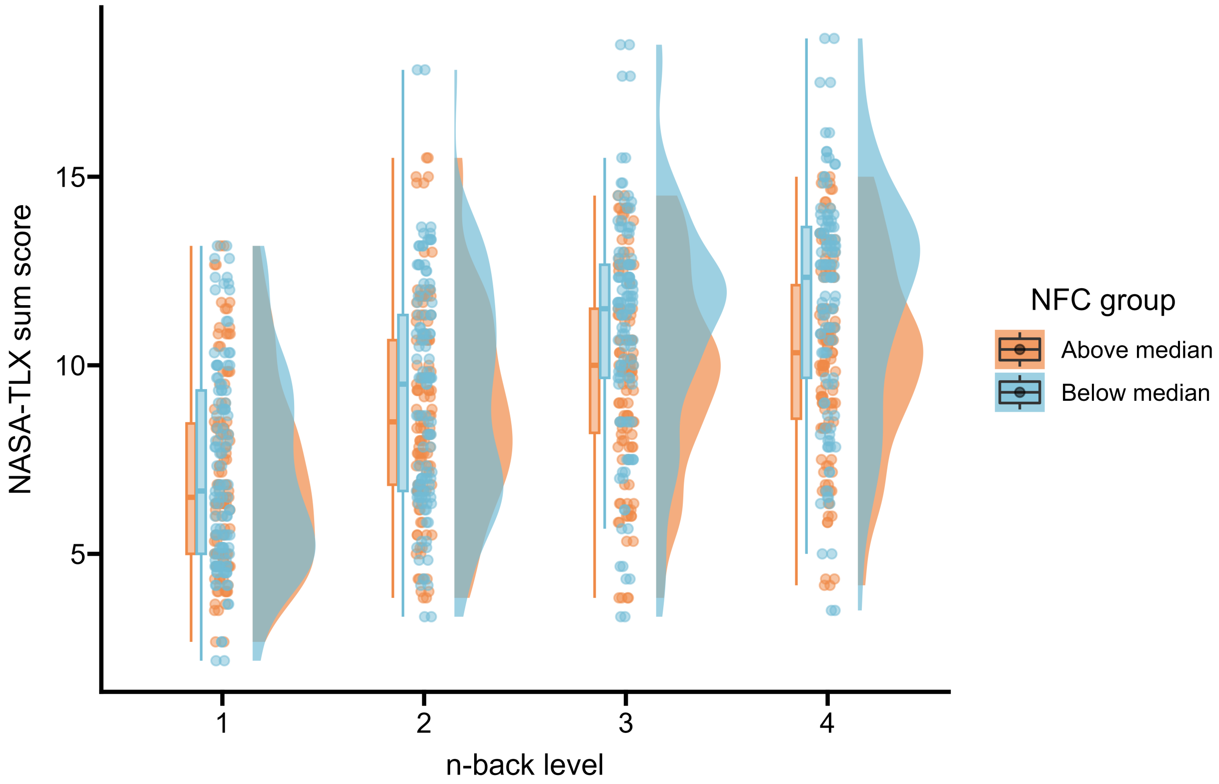

Figure S.3. NASA-TLX sum scores for each n-back level. Colours indicate Need for Cognition (NFC) score above or below the median. N = 116. Figure available at osf.io/vnj8x/, under a CC-BY-4.0 license.

Hypothesis 3c: Participants with higher NFC scores have lower aversiveness ratings for 2- and 3-back but higher higher aversiveness ratings for 1-back than participants with lower NFC scores.

ANOVA:

Main effect level: $F(1, 114) = 10.21$, $p = .002$, $\hat{\eta}^2_G = .034$, 90% CI $[.000, .105]$,

Main effect NFC group: $F(1, 114) = 8.43$, $p = .004$, $\hat{\eta}^2_G = .043$, 90% CI $[.003, .119]$, $\mathrm{BF}_{10} = 14.26$

Interaction: $F(1, 114) = 2.59$, $p = .110$, $\hat{\eta}^2_G = .009$, 90% CI $[.000, .058]$

Contrast for main effect level:

Table S.14
Paired contrast for the rmANOVA comparing the influence of Need for Cognition group and n-back level on difference scores of aversiveness ratings.

Contrast	Estimate	SE	df	t	p	BF_{10}	η^2_p	$95\% CI$
1-2 - 2-3	1.27	0.40	114.00	3.20	0.002	5.49	0.08	[0.02, 1.00]

Note. The column Contrast contains the n of the n-back levels. SE = standard error, df = degrees of freedom, t = t-statistic, p = p-value, CI = confidence interval.

Contrast for main effect NFC group:

Table S.15
Paired contrast for the rmANOVA comparing the influence of Need for Cognition (NFC) group and n-back level on difference scores of aversiveness ratings.

Contrast	Estimate	SE	df	t	p	BF_{10}	η^2_p	$95\% CI$
High NFC - Low NFC	1.44	0.50	114.00	2.90	0.004	14.26	0.07	[0.01, 1.00]

Note. SE = standard error, df = degrees of freedom, t = t-statistic, p = p-value, CI = confidence interval.

Exploratory analysis with NFC groups by median split

ANOVA:

Main effect level: $F(2.01, 229.39) = 67.39$, $p < .001$, $\hat{\eta}^2_G = .295$, 90% CI $[.228, .354]$, 2.70×10^{30}

Main effect NFC group: $F(1, 114) = 2.63$, $p = .108$, $\hat{\eta}^2_G = .007$, 90% CI $[.000, .053]$, 2.95×10^{-1}

Interaction: $F(2.01, 229.39) = 3.24$, $p = .041$, $\hat{\eta}^2_G = .020$, 90% CI $[.000, .044]$

Contrasts for main effect level:

Table S.16

Paired contrast for the rmANOVA comparing the influence of Need for Cognition group and n-back level on subjective values.

Contrast	Estimate	*SE*	*df*	*t*	*p*	BF_{10}	η^2_p	95%*CI*
1 - 2	0.03	0.02	114.00	2.03	0.184	0.66	0.03	[0.00, 1.00]
1 - 3	0.15	0.03	114.00	5.77	<.001	117,986.95	0.23	[0.12, 1.00]
1 - 4	0.33	0.03	114.00	9.70	<.001	1.47×10^{13}	0.45	[0.34, 1.00]
2 - 3	0.11	0.02	114.00	5.55	<.001	67,167.07	0.21	[0.11, 1.00]
2 - 4	0.30	0.03	114.00	10.22	<.001	4.31×10^{14}	0.48	[0.37, 1.00]
3 - 4	0.19	0.03	114.00	7.30	<.001	1.68×10^{8}	0.32	[0.21, 1.00]

Note. The column Contrast contains the n of the n-back levels. SE = standard error, *df* = degrees of freedom, t = t-statistic, p = p-value, CI = confidence interval.

Contrasts for the interaction:

Table S.17

Paired contrast for the rmANOVA comparing the influence of Need for Cognition group and n-back level on subjective values.

Contrast	Level	Estimate	SE	df	t	p	BF_{10}	η_p^2	$95\% CI$
high - low	1	-0.05	0.03	114.00	-1.50	0.136	0.54	0.02	[0.00, 1.00]
high - low	2	0.02	0.03	114.00	0.72	0.472	0.25	4.54e-03	[0.00, 1.00]
high - low	3	0.05	0.04	114.00	1.28	0.203	0.41	0.01	[0.00, 1.00]
high - low	4	0.11	0.05	114.00	2.13	0.036	1.48	0.04	[0.00, 1.00]

Note. The column Contrast contains the groups above (high) and below (low) the Need for Cognition score median. The column Level contains the n of the n-back levels. SE = standard error, df = degrees of freedom, t = t-statistic, p = p-value, CI = confidence interval.

Exploratory analysis with NFC as a continuous covariate

ANOVA (NFC scores standardized):

Table S.18

Result of the rmANOVA regarding the predictive power of the n-back level on the subjective values when including Need for Cognition scores as a centered continuous covariate.

	Sum Sq	*df*	error Sum Sq	den *df*	*F*	*p*	η_p^2	95%*CI*
Intercept	301.49	1.00	5.44	114.00	6,322.13	<.001	0.98	[0.98, 1.00]
NFC	0.21	1.00	5.44	114.00	4.34	0.039	0.04	[0.00, 1.00]
n-back level	7.83	3.00	13.28	342.00	67.24	<.001	0.37	[0.30, 1.00]
NFC x n-back level	0.44	3.00	13.28	342.00	3.78	0.011	0.03	[0.00, 1.00]

Note. NFC = Need for Cognition. SE = standard error, *df* = degrees of freedom, F = F-statistic, p = p-value, η_p^2 = partial eta squared, CI = confidence interval.

Estimated marginal means of the linear trends:

Table S.19

Paired contrast slope analysis as a follow up for the rmANOVA regarding the predictive power of the n-back level on the subjective values when including Need for Cognition scores as a centered continuous covariate.

n-back level	Estimate	SE	*df*	t	p
1 - 2	-0.03	0.03	456.00	-1.11	0.68
1 - 3	-0.03	0.03	456.00	-1.27	0.58
1 - 4	-0.09	0.03	456.00	-3.22	0.01
2 - 3	0.00	0.03	456.00	-0.16	1.00
2 - 4	-0.06	0.03	456.00	-2.10	0.15
3 - 4	-0.05	0.03	456.00	-1.95	0.21

Note. The column n-back level contains the n of the task levels. SE = standard error, *df* = degrees of freedom, t = t-statistic, p = p-value.

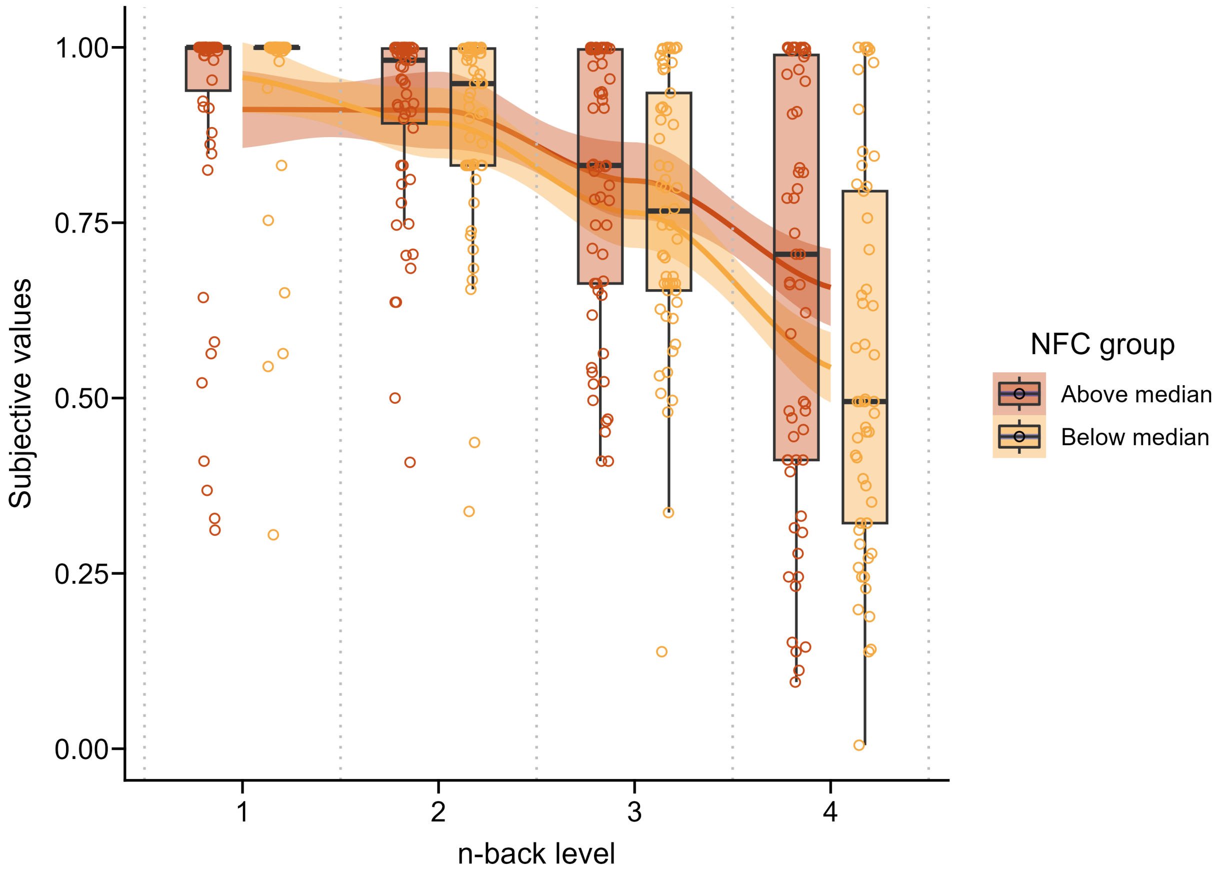

Figure S.4. Subjective values per n-back level for participants with Need for Cognition (NFC) scores above and below the median. $N = 116$. The scatter has a horizontal jitter of 0.2. Smoothing of conditional means with Loess method. Figure available at osf.io/vnj8x/, under a CC-BY-4.0 license.
